# 3D cell culture models in research: applications to lung cancer pharmacology

**DOI:** 10.3389/fphar.2024.1438067

**Published:** 2024-09-23

**Authors:** Nathan Vella, Anthony G. Fenech, Vanessa Petroni Magri

**Affiliations:** Department of Clinical Pharmacology and Therapeutics, Faculty of Medicine and Surgery, University of Malta, Msida, Malta

**Keywords:** 3D cell culture, lung cancer, tumour microenvironment, pre-clinical models, drug screening, drug development, precision oncopharmacology

## Abstract

Lung cancer remains one of the leading causes of cancer-related mortality worldwide, necessitating innovative research methodologies to improve treatment outcomes and develop novel strategies. The advent of three-dimensional (3D) cell cultures has marked a significant advancement in lung cancer research, offering a more physiologically relevant model compared to traditional two-dimensional (2D) cultures. This review elucidates the various types of 3D cell culture models currently used in lung cancer pharmacology, including spheroids, organoids and engineered tissue models, having pivotal roles in enhancing our understanding of lung cancer biology, facilitating drug development, and advancing precision medicine. 3D cell culture systems mimic the complex spatial architecture and microenvironment of lung tumours, providing critical insights into the cellular and molecular mechanisms of tumour progression, metastasis and drug responses. Spheroids, derived from commercialized cell lines, effectively model the tumour microenvironment (TME), including the formation of hypoxic and nutrient gradients, crucial for evaluating the penetration and efficacy of anti-cancer therapeutics. Organoids and tumouroids, derived from primary tissues, recapitulate the heterogeneity of lung cancers and are instrumental in personalized medicine approaches, supporting the simulation of *in vivo* pharmacological responses in a patient-specific context. Moreover, these models have been co-cultured with various cell types and biomimicry extracellular matrix (ECM) components to further recapitulate the heterotypic cell-cell and cell-ECM interactions present within the lung TME. 3D cultures have been significantly contributing to the identification of novel therapeutic targets and the understanding of resistance mechanisms against conventional therapies. Therefore, this review summarizes the latest findings in drug research involving lung cancer 3D models, together with the common laboratory-based assays used to study drug effects. Additionally, the integration of 3D cell cultures into lung cancer drug development workflows and precision medicine is discussed. This integration is pivotal in accelerating the translation of laboratory findings into clinical applications, thereby advancing the landscape of lung cancer treatment. By closely mirroring human lung tumours, these models not only enhance our understanding of the disease but also pave the way for the development of more effective and personalized therapeutic strategies.

## 1 Introduction

Lung cancer is a worldwide leading cause of cancer-related deaths, claiming a global 1.8 million fatalities per year (Sung et al., 2021; Adjei, 2019). It is a heterogeneous disease which has proven difficult to treat, and provides a formidable challenge to healthcare and research workers alike. The two main lung cancer subtypes are non-small cell lung cancer (NSCLC) and small cell lung cancer (SCLC). NSCLC is the more common subtype, comprising about 85% of lung cancers and claiming a 5-year survival rate of only 15%. NSCLC is sub-classified into adenocarcinoma, squamous cell carcinoma (SCC) and large cell carcinoma (LCC). Strongly associated with smoking, SCLC is subdivided into pure small cell carcinoma and combined small cell carcinoma, and is characterized by its rapid growth and early metastasis, having an estimated survival rate of 6.2% (Rudin et al., 2021; Liang et al., 2022).

Treatment of lung cancer has posed a global challenge. The World Health Organization had long advocated for minimizing the incidence rate through the avoidance of risk factors, the main ones being tobacco and workplace related hazards such as chemicals and asbestos (World Health Organization, 2023). Current therapies mainly include surgery, chemotherapy, radiation therapy, targeted therapy (e.g., EGFR tyrosine kinase inhibitors (TKI), KRAS-G12C therapies and ALK gene fusions) and immunotherapy (e.g., anti-PD-L1 immunotherapy). While surgery is limited to early-stage tumours, standard chemotherapy and radiation monotherapies have reached a therapeutic limit resulting from severe side-effects and the development of therapy resistance (Huang et al., 2017; Yegya-Raman et al., 2018). Following their recent emergence, antibody drug conjugates (ADC) and immune checkpoint inhibitors (ICI) enable tumour cell targeting and are currently undergoing clinical trials (Desai et al., 2022; Colombo and Rich, 2022; Genova et al., 2022). However, the survival rates for lung cancer remain exceptionally low, in conjunction with a drug development pipeline which has been repeatedly marked with a very high drug attrition rate at the clinical stage (Huo et al., 2020). These outcomes emphasize the urgent need for safe and effective novel therapies to target lung cancer.

Advancements in lung cancer research have increasingly focused on developing more physiologically relevant models to better understand the complexities of the disease and to improve drug discovery efforts. Traditional 2D cell cultures, while foundational, fall short in replicating the intricate tumour microenvironment (TME) and cellular interactions that characterize lung cancers *in vivo*. In response, the latest 3D cell culture models, including spheroids, organoids, tumoroids and microfluidic devices, have emerged as powerful tools that more accurately mimic the architecture, gene expression and drug responses observed clinically. In order to develop biomimicking lung cancer models *in vitro*, it is necessary to understand both the histology and function of the normal human lung and the intricacies of the lung TME consisting of complex interactions and responses between various cell types and the extracellular matrix (ECM). Hence, this article provides a solid foundation to the lung microenvironment and explores cutting-edge 3D lung cancer models, focusing on their potential to revolutionize lung cancer pharmacology research by providing more predictive and reliable platforms for preclinical drug testing and personalized medicine.

## 2 Normal human lung histology

The normal human respiratory framework comprises various cell types which orchestrate lung functionality by creating an optimal environment for gas exchange and activate several protective mechanisms. One of the most abundant cell types in the human airway epithelium are the pseudostratified ciliated columnar epithelial cells (or ciliated cells). Goblet cells and basal epithelial cells also line the tracheal and bronchial epithelium. This epithelium is covered by a layer of mucus mainly consisting of 95% water, 2%–5% mucins (glycoproteins), salts and other proteins and cell fragments (Murgia et al., 2017). Goblet cells secrete mucin granules at their apical surface and therefore help maintain moistness by supporting the formation of the mucus layer which protects the airway from dust, bacteria and other contaminants via the mucociliary escalator (Ganesan et al., 2013). Basal cells are multipotent stem cells involved in epithelial cell renewal, particularly of ciliated cells, and are located at the basement membrane where they support the attachment of ciliated cells. Additionally, basal cells interact with neurons and immune cells and can also be found in the bronchi and down to the terminal bronchioles in lower numbers (Evans et al., 2001; Rackley and Stripp, 2012).

In the respiratory bronchioles, goblet cells are replaced by non-ciliated club (Clara) cells. Club cells constitute around 9% of the total lung epithelium and are present within the lower parts of the airway beginning at the terminal bronchioles (Khan and Lynch, 2023; Rokicki et al., 2016). Protruding above the level of neighbouring cells, club cells possess secretory granules which secrete various lipoproteins (such as Clara cell secretory protein, CCSP or CC10) and surfactant proteins. Therefore, club cells seem to have a homeostatic role with regards to surfactant fluid (Antunes et al., 2013; Lowe and Anderson, 2015).

Brush cells, present from the nasal cavity to the bronchioles, are thought to act as chemoreceptors, though their exact function is unclear (Brody, 2005; Khan and Lynch, 2023; Hollenhorst et al., 2020). Neuroendocrine cells, containing neurosecretory granules, secrete polypeptide hormones and neuropeptides (trigger immune responses) in response to environmental stimuli. Making up 1%–3% of the epithelial cell layer, neuroendocrine cells are considered to be the only innervated cells in the human lung epithelium, possibly controlling goblet cell and submucosal gland activity (Noguchi et al., 2020; Rokicki et al., 2016; Branchfield et al., 2016; Khan and Lynch, 2023).

**FIGURE 1:**
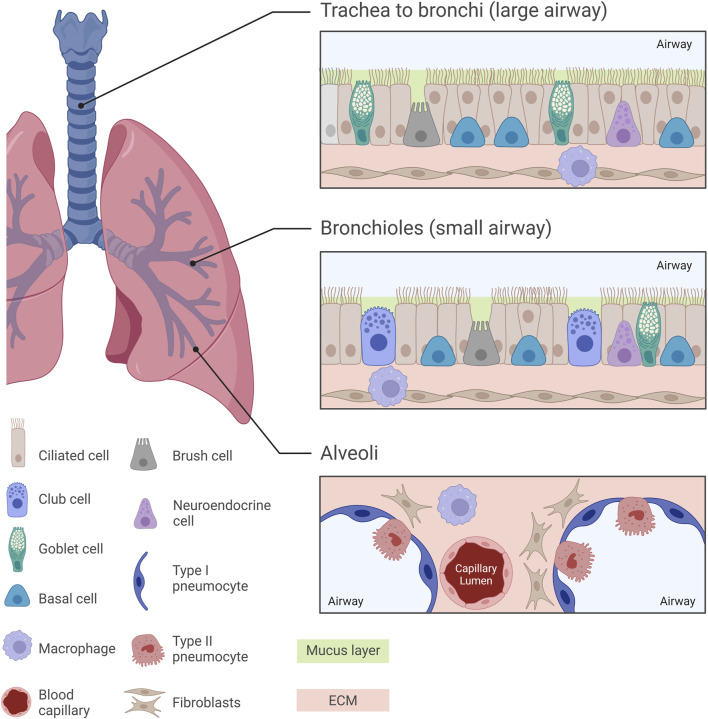
Normal pulmonary histology at different regions of the human lung. Diverse histological features can be observed across different regions of the human lung, from the trachea towards the alveoli, with each region exhibiting specialized cellular arrangements crucial for respiratory function. The trachea, bronchi and bronchioles are characterized by a pseudostratified ciliated columnar epithelium, while the intricate alveolar structures are lined by Type I and Type II pneumocytes. In the small airway, mucus-secreting goblet cells are replaced by surfactant-producing club cells, paralleled with a decrease in the thickness of the mucus layer lining the airway epithelium. Understanding these histological nuances is essential for unravelling the complexities of lung pathology and developing pharmacological *in vitro* models which replicate the *in vivo* scenario.

Type I and Type II pneumocytes line the alveoli: Type I facilitate gas exchange, while Type II are involved in surfactant production, immune cell modulation and stem cell activity (Ruaro et al., 2021; Glisinski et al., 2020). The lungs are safeguarded by a variety of immune cells such as macrophages and dendritic cells which act by phagocytosing pathogens and presenting antigens, respectively (Khan and Lynch, 2023; Harkema et al., 2018). Lung fibroblasts are integral components of the ECM, modulating tissue architecture and elasticity through ECM synthesis and remodelling (White, 2015; Chua and Laurent, 2006). Histological features of the healthy human lung are illustrated in Figure 1.

## 3 The lung tumour microenvironment

**FIGURE 2:**
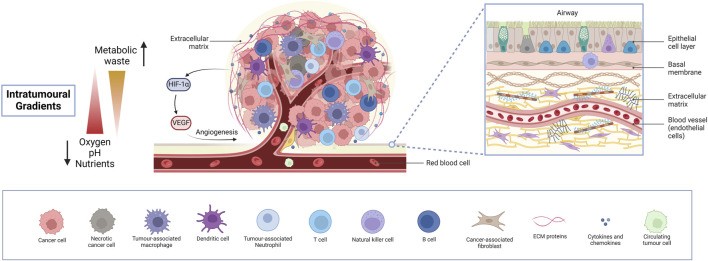
The lung tumour microenvironment. An insight into the intricate heterogeneous cellular and molecular landscape of the lung TME, exhibiting diverse cell types, intra-tumoural gradients and activation of angiogenesis. Cancer cells, together with various immune cells, fibroblasts, and endothelial cells lining blood vessels and several ECM components orchestrate a dynamic interplay driving tumour progression, metastasis and therapy resistance, while limiting drug accessibility. Necrotic areas are fundamentally responsible for the activation of angiogenesis, paving the way for extensive tumour growth. Understanding these complex interactions is crucial for developing lung cancer models for preclinical pharmacological research to effectively target lung cancer and study drug responses. (Abbreviations: TME: tumour microenvironment, ECM: extracellular matrix).

The idea of the TME was first coined in 1979 by Lord et al. who studied the interactions between different cells in a tumour (Lord et al., 1979). Apart from tumour cells, the TME consists of several cell types, mainly immune cells, fibroblasts, endothelial cells and cancer stem cells (CSC) (Li and Qiao, 2022; Anderson and Simon, 2020; Wu F. et al., 2021). These cells interact with tumour cells and secrete signalling molecules and ECM components, creating a supportive environment for tumour growth and progression. The ECM consists of both fibrous and multi-adhesive proteins, such as collagen and fibronectin, respectively. It serves to support the spatial arrangement of multiple cell types within the TME all of which communicate with each other and with the ECM to promote tumour growth, immunosuppression and therapy resistance (Labani-Motlagh et al., 2020). Cell-ECM interactions alter structural components to form a physical barrier to the TME, limiting drug accessibility. The lack of homogeneity and limited vasculature penetrability present another barrier to tumour drug delivery (Pinto et al., 2020). In short, the lung TME is a remodelled niche, exploiting every cell type to support tumour growth and metastasis (Altorki et al., 2019). As an area of growing interest, a deep understanding of the TME is crucial for creating accurate biomimetic 3D models. The general cell types and features of the lung TME are illustrated in Figure 2.

### 3.1 The lung cancer immune landscape

The immune landscape of lung cancer, particularly non-small cell lung cancer (NSCLC), is a complex and heterogeneous environment that plays a crucial role in the disease’s progression and response to therapies. Indeed, recent studies using single-cell sequencing technologies have identified distinct immune signatures associated with different subtypes of lung cancer, specifically lung adenocarcinoma and lung squamous cell carcinoma (Wang et al., 2022).

A total of 13 tumour immune cell populations have been identified in NSCLC, including macrophages, neutrophils, dendritic cells (DC), natural killer (NK) cells, T cells and B cells, having complex roles and accompanied by immunosuppressive mechanisms (Labani-Motlagh et al., 2020; Madeddu et al., 2022). While the types of NSCLC tumour-infiltrating immune cells are associated with clinical outcomes, strategies aimed at modulating immune cell function are being actively explored (Balážová et al., 2023; Tiwari et al., 2022). Most studies suggest a macrophage-dominant lung cancer tumour-infiltrating immune cell landscape, although other studies exhibited a neutrophil or T cell dominant one (Tamminga et al., 2020; Balážová et al., 2023; Kargl et al., 2017; Liu et al., 2017; Stankovic et al., 2019; Evans et al., 2021). Discrepancies across studies may point towards the extreme heterogeneity of lung cancer amongst different populations. Research on the immune landscape of SCLC is still very lacking.

Tumour-associated macrophages (TAMs), which have been recently described as a unique phenotypic state, play a role in driving the inflammatory response within tumours and are associated with metastatic activation, drug resistance and poor prognosis (Balážová et al., 2023). TAM formation is activated by TME stimuli, including the presence of cancer-associated fibroblasts (CAF) and hypoxia (Tan et al., 2021; Liu J. et al., 2021; Xu et al., 2022; Garnique and Machado-Santelli, 2023; Madeddu et al., 2022).

Macrophages may exist in two major polarisation states: M1 (classically activated; pro-inflammatory) can enhance antitumour immunity (Barrera et al., 2023), while M2 (alternatively activated; anti-inflammatory) secrete immunosuppressive cytokines such as IL-10 and TGF-β and induce T_reg_ expansion, contributing to reduced tumour immunity (Balážová et al., 2023; Bremnes et al., 2011). Additionally, a third undifferentiated subtype, M0, has been recently identified by Hickman et al. (2023). These subtypes have been shown to provide distinct immune signatures that can differentiate lung adenocarcinoma from lung squamous cell carcinoma. Notably, the dominant macrophage subtype in adenocarcinoma was identified as the FABP4-expressing M1 subtype, whereas SPP1-expressing M2 macrophages were predominant in lung squamous cell carcinoma (Wang et al., 2022). While the different subtypes cannot be identified morphologically, a simple immunohistochemistry method based on the high expression of IL12 and CCR7 in M1 and CD163 and ALOX15 in M2 subtypes has been described (Zheng et al., 2020).

T cells constitute a similarly important and highly abundant immune cell type in lung tumours, comprising approximately 47% of all CD45^+^ immune cells. CD4^+^ T helper cells and CD8^+^ cytotoxic T cells are the predominant subsets, followed by a smaller population of double-negative CD4^−^ CD8^−^ T cells (Stankovic et al., 2019). CD8^+^ T cells are considered the primary effectors of antitumor immunity, capable of directly killing cancer cells upon recognition of tumour antigens presented by MHC class I molecules. CD4^+^ T cells can differentiate into various helper and regulatory subsets that modulate the immune response. Th1 cells secrete IFN-γ and promote CD8^+^ T cell cytotoxicity, while Th2 cells support humoral immunity. Regulatory T cells (T_regs_) suppress effector T cell function and are associated with a worse prognosis in lung cancer (Bremnes et al., 2011; Zheng et al., 2017).

B cells comprise about 16% of the CD45^+^ population in lung tumours. They can contribute to antitumor immunity through antibody production, antigen presentation, and cytokine secretion. However, their role in lung cancer is complex, with some studies linking high B cell infiltration to improved survival (Barrera et al., 2023) and others to worse outcomes (Bremnes et al., 2011).

Natural killer (NK) cells make up about 4.5% of the lung tumour immune infiltrate (Stankovic et al., 2019). They can directly lyse tumour cells and secrete cytokines such as IFN-γ that enhance antitumor immunity. Depletion of NK cells has been shown to promote lung tumour growth in mouse models (Barrera et al., 2023), suggesting that they play a protective role.

Dendritic cells are less abundant in lung tumours, comprising only about 2.1% of CD45^+^ cells. However, they play a crucial role in antigen presentation to T cells. Plasmacytoid dendritic cells secrete type I interferons and promote Th1 responses, while conventional dendritic cells can cross-present tumour antigens to CD8^+^ T cells (Bremnes et al., 2011).

The spatial organization of immune cells within tumours modulates the interactions between themselves, as well as with the surrounding tissue. This may have implications in tumour progression, proliferative activity, treatment responses and survival, and the challenge to reproduce this environment accurately in a 3D cell culture model may be best addressed by *ex vivo* 3D setups. For example, Parra et al. (2023) used a 23-marker tumour immunoprofiling panel on NSCLC tumour cells, and reported that CD3^+^ CD8^+^ cytotoxic T-cells were the most abundant immune cells in both adenocarcinoma and squamous cell carcinoma, but were relatively distant from malignant cells. In contrast, T-cells expressing PD-L1, B7-H3, B7-H4, IDO-1, and OX40, were less abundant, but were located closer to the malignant cells, suggesting that the distance from malignant cells and distribution patterns both play a role in cancer.

High densities of TAMs are well known to be associated with poor outcomes in many types of cancer. Interestingly, cancer cells undergoing apoptosis have been reported to be located closer to pro-inflammatory M1-polarized macrophages than to anti-inflammatory M2-polarized macrophages, while the reverse was true for tumour cells which were positive for the Ki67 proliferative marker (Zheng et al., 2020). Spatial cellular organisation and immune cell interactions may also influence immunotherapy outcomes. For example, the presence of high spatial niches of T cells, and macrophages in NSCLC adenocarcinomas, increases clonal neoantigen burden, potentially increasing the response of such immune-hot tumours to immunotherapy (Parra et al., 2023).

After studying samples from 120 NSCLC adenocarcinoma patients with disease stages ranging from I to III Barua et al. (2018) reported tumour cell and regulatory T-cell (T_reg_) interactions to be significantly associated with worse survival, while the co-presence of cytotoxic CD8^+^ T lymphocytes resulted in better survival. Infiltration of T_reg_ cells into core tumour regions may therefore be an independent predictor of worse overall survival in NSCLC, with the effect being mitigated by co-infiltration of CD8^+^ cytotoxic T cells. In addition to the influence of immune cells, the tumour vasculature is an important contributor to tumour proliferation rates. This makes the contributory roles of immune cells more difficult to dissect. Enfield et al. (2024) studied the proliferation rates of lung squamous cell carcinoma, based on the differential cell densities of tumour infiltrating lymphocytes (TILs, T cells and B cells), macrophages, and neutrophils within the tumour nest (T) or stroma (S). The lowest proliferative tumours were found to be those with the greatest nest and stromal neutrophil infiltration. Interestingly, these same tumours exhibited the greatest distances between each tumour cell and its nearest endothelial cell, suggesting that the combination of neutrophil activity and reduced oxygen supply jointly contributed to the lower proliferation.

### 3.2 Immune evasion strategies in lung tumours and implications for treatment

Lung tumours employ various molecular mechanisms to evade the immune system, and these mechanisms also influence immunotherapy treatment. There are three overarching mechanisms that tumours bring into play for immune evasion.

*Upregulation of immune checkpoint molecules:* One of the most well-studied mechanisms of immune evasion in lung cancer is the upregulation of immune checkpoint molecules, such as programmed death-ligand 1 (PD-L1). PD-L1 expressed on lung cancer cells binds to the PD-1 receptor on T cells, triggering an inhibitory signal that leads to T cell exhaustion and dysfunction. High expression of PD-L1 is often associated with poor prognosis in lung cancer patients.

*Impairment of antigen presentation:* Lung cancer cells can evade immune detection by downregulating or altering the expression of major histocompatibility complex (MHC) class I molecules, which are responsible for presenting tumour-specific antigens to cytotoxic T cells. This process, known as “MHC class I downregulation,” has been observed in up to 90% of lung tumours (Qin et al., 2016). This can be brought about through various mechanisms including; (a) loss of heterozygosity (LOH) at the *HLA* locus, leading to the complete loss of *HLA* expression (Anichini et al., 2020), (b) defects in the antigen processing machinery, such as downregulation of the transporter associated with antigen processing (TAP) (Gupta et al., 2023), (c) epigenetic silencing of genes involved in antigen presentation, such as *NLRC5*, a key regulator of MHC class I-dependent immune responses (Kobayashi and van den Elsen, 2012).

*Induction of an immunosuppressive TME:* Lung tumours create an immunosuppressive microenvironment by recruiting and activating various immunosuppressive cells, such as T_regs_ and myeloid-derived suppressor cells (MDSC). These cells secrete inhibitory cytokines such as IL-10 and TGF-β, which suppress the function of effector T cells. Additionally, lung cancer cells can induce the expression of indoleamine 2,3-dioxygenase (IDO), an enzyme that catabolizes tryptophan, leading to T cell anergy and apoptosis (Salehi-Rad and Dubinett, 2019).

The molecular mechanisms of immune evasion in lung cancer have important implications for immunotherapy, the aim of which is to enhance the immune system’s ability to detect and destroy cancer cells. One of the primary therapeutic modalities involves the use of immune checkpoint inhibitors, which block proteins that inhibit immune responses. Drugs such as pembrolizumab and nivolumab which block programmed cell death protein 1 (PD-1), a T cell expressed receptor, have been successfully used in patients with metastatic NSCLC with high PD-L1 expression and have demonstrated increased survival rates. Other therapeutic approaches have targetted the inhibition of PD-L1 which normally interacts with its receptor PD-1 on T cells and signals to inhibit T cell receptor (TCR)-mediated activation of IL-2 production and T cell proliferation. Examples of such drugs are atezolizumab and durvalumab, both of which are used for advanced metastatic NSCLC. Ipilimumab is an inhibitor of cytotoxic T-lymphocyte associated protein 4 (CTLA-4). This protein is a receptor that is constitutively expressed on T_regs_ and CD4^+^/CD8^+^ conventional T cells. When activated by the B7 ligand, it is upregulated in conventional T cells and signals to deactivate them. By inhibiting CTLA-4, ipilimumab enhances T cell activation and proliferation, enhancing the immune response against cancer cells. It is may be used in combination with nivolumab for advanced NSCLC, providing a synergistic effect that improves treatment outcomes (Hellmann et al., 2019).

The contributions of immune cells within a tumour may be further confounded by the mechanical properties of the ECM. For example, changes in stiffness arising from matrix remodelling through the action of various enzymes such as matrix metalloproteinases (MMP) can facilitate immune cell movement or, conversely, contribute to a more immunosuppressive environment. It has been shown that T cells cultured in high-density collagen matrices exhibit diminished cytotoxic activity and reduced production of key cytokines such as IFN-γ, which are vital for an effective immune response (Du et al., 2024). In NSCLC, osteopontin (OPN), a multifunctional extracellular matrix protein, is associated with the amplification of the checkpoint protein PD-L1 via the NF-κB pathway, therefore serving as a tactical manoeuvre employed by the tumour to elude immune surveillance and annihilation (Li et al., 2021a).

Combining immunotherapy with chemotherapy or targeted therapies has become a standard approach in managing lung cancer. For instance, the combination of pembrolizumab with chemotherapy has shown superior efficacy compared to chemotherapy alone in first-line treatment settings. Such combinations leverage the synergistic effects of different treatment modalities, enhancing overall therapeutic outcomes (Dang et al., 2016; Ruiz-Cordero and Devine, 2020; Alexander et al., 2020; Naratornsirakul et al., 2024).

### 3.3 Cancer-associated fibroblasts

Fibroblasts are one of the most common cell types present in the lung tumour stroma, of which three subtypes have been identified in NSCLC (Hu H. et al., 2021). In the presence of cancer cells, fibroblasts phenotypically switch to cancer-associated fibroblasts (CAF), marked by a decrease in expression and functional pro-tumour alterations of p53 (Arandkar et al., 2018). CAFs seem to increase therapy resistance in NSCLC, including that of EGFR TKIs, by modulating signalling pathways, activating receptors and via cross-talks with cancer cells. CAFs co-cultured with lung cancer cells were found to increase proliferation and tumour cell survival, induce epithelial-mesenchymal transition (EMT), maintain CSC stemness and promote chemoresistance (Nakamura et al., 2019; Kanaji et al., 2017; Wang et al., 2019; You et al., 2019; Meyerholz et al., 2018; Worrell and MacLeod, 2021). Release of chemokines and cytokines by CAFs was shown to promote ECM remodelling, alter the immune landscape and induce autophagy resulting in the recovery of lung cancer cells from radiation-induced damage, together with elevating the tumour metastatic potential via paracrine signalling involving STAT3 (Shien et al., 2017; Wang et al., 2017; Suzuki et al., 2022; Wong et al., 2022; Zhang H. et al., 2021). Their increased expression of hypoxia-inducible factor 1-alpha (HIF-1α) allows CAFs to survive and modulate hypoxic environments, further stimulating NSCLC therapy resistance (Chen et al., 2021). An improved understanding of the TME has initiated the discovery of anti-cancer therapies targeted towards CAFs aiming to minimize lung cancer resistance and other oncogenic properties (Chen et al., 2021; Zhang H. et al., 2021; Papait et al., 2022).

### 3.4 Tumour endothelial cells

Tumour endothelial cells within the TME orchestrate neo-vascularisation, forming blood vessels for the transport of oxygen, nutrients and metabolic waste. These cells support tumour growth and provide a vascular system for the release of tumour cells in the early stages of metastasis (Hida et al., 2022). Upregulation of HIF-1α in hypoxic conditions promotes increases in pro-angiogenic factors, namely, vascular endothelial growth factor (VEGF), known to be overexpressed in many lung cancers, causing an increase in tumour endothelial cell and tumour cell proliferation and angiogenesis (Wieleba et al., 2022; Frezzetti et al., 2017; Becker et al., 2023; Zhao Y. et al., 2022). Unlike traditional 2D cell cultures, *VEGF* gene expression was found to be regulated by HIF-1α in lung cancer 3D models, similar to what is observed *in vivo* (Onodera et al., 2023). Anti-VEGF therapies are emerging as one of the latest trends in targeting lung cancer (Frezzetti et al., 2017; Yang et al., 2017; Jeong et al., 2020; Seitlinger et al., 2022).

### 3.5 Oxygen, pH and nutrient gradients

A hallmark in all solid tumours is the decreased access of oxygen within deep cell layers due to the lack of neighbouring blood vessels (Hida et al., 2022). Oxygen is known to have a diffusion gradient of approximately 100–200 μm, causing an oxygen gradient in solid tumours including lung cancers. In NSCLC, intra-tumoural oxygen concentrations have been reported to be at around 2%, compared to 5.6% in normal lung tissue. The ability of tumours to sustain hypoxia arises from a phenomenon known as the Warburg effect which induces a metabolic switch in cancer cells from oxidative phosphorylation to aerobic glycolysis (Pinto et al., 2020). This is driven by the upregulation of HIF-1α, promoting survival under hypoxic conditions leading to NSCLC aggressiveness and resistance to target therapy and chemotherapy (Liu C. et al., 2021; Ziółkowska-Suchanek, 2021; Lu et al., 2020). Hypoxic regions of NSCLC are known to be characterized by a unique immune-suppressing landscape (Zhang C. et al., 2021). The potential of hypoxia-activated prodrugs for lung cancer therapy is currently being investigated (Li et al., 2021b; Singleton et al., 2021).

Solid tumours are additionally characterized by nutrient and metabolic waste gradients. The conversion of glucose to lactate causes lactic acid release, with a notable accumulation within tumours. The increase in intra-tumoural lactic acid is accompanied by an increased export to the TME via monocarboxylate transporters. This drop in pH in the TME and within solid tumours causes immune cell-activated oncogenic properties, including angiogenesis and drug resistance, while inhibiting drug permeability by ion trapping (Li and Qiao, 2022; Gao et al., 2022). Furthermore, the distances of cells from blood and lymph vessels gives rise to nutrient gradients, inducing distinct cellular metabolic profiles and increased immunosuppression (García-Cañaveras et al., 2019; Singleton et al., 2021).

## 4 *In vitro* lung cancer models used for pharmacological studies

From the development of the first cell line in 1951, the popularity of *in vitro* cell-based studies has dramatically increased and multiple cell lines have become commercially available, together with their clinical and molecular classifications (Scherer et al., 1953). Although these have provided the basis for *in vitro* laboratory studies on the molecular pathology and pharmacology of these cancers, 2D culture models have often fallen short of providing a suitable representative model of an *in vivo* tumour. Consequently, the use of 2D cultures suffers from limitations to translational relevance, known to be a major cause of the low success rate of clinical trials (Huo et al., 2020).

Lung cancer 3D culture models develop other key features which replicate those found within the TME which have already been well reviewed. These include similar cell-cell and cell-ECM contacts through the expression of cadherins, ECM formation, intra-tumoural gradients and cell layer organization. At diameters greater than 400 μm 3D cancer models develop necrotic, quiescent and proliferative cell layers, replicating the *in vivo* scenario (Pinto et al., 2020; Rozenberg et al., 2021; Shie et al., 2023; Hamilton and Rath, 2019). More complex models can simulate tumour vascularization and perfusion across blood vessels, air exposure and biophysiological characteristics.

**FIGURE 3:**
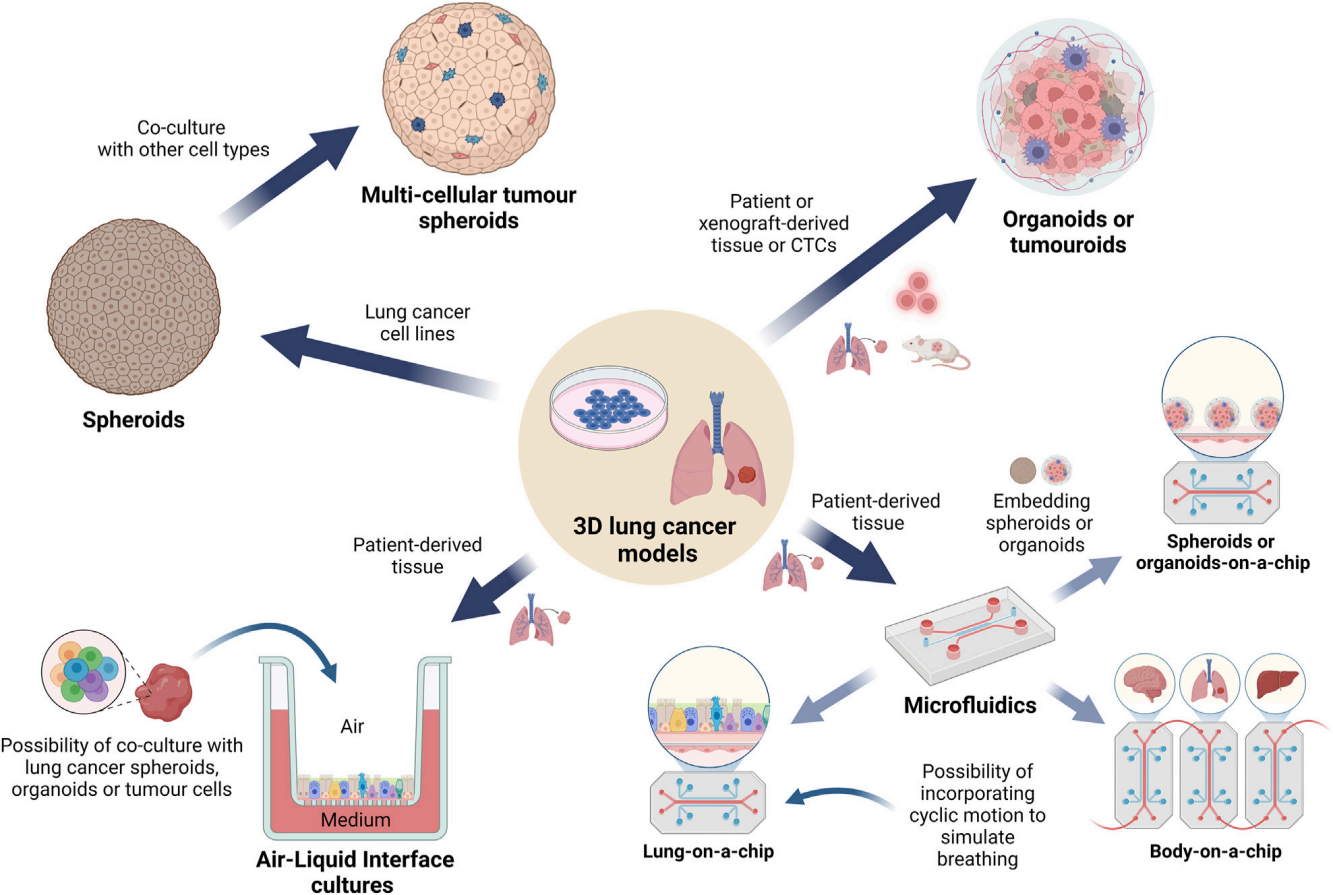
Overview of the 3D cell culture models currently used in lung cancer pharmacology research. A graphical representation depicting the main types of 3D lung cancer models which are being used for *in vitro* lung cancer pharmacological studies. Spheroids and organoids are the most common types of cell cultures, followed by tumouroids, while the more complex microfluidic models are used to study specific cellular interactions, angiogenesis or metastasis to other organs. These models may incorporate biomaterials generated via bioprinting technologies while maintaining streamlined workflows. Use of ALI conditions has gained increasing interest in simulating the lung environment for drug testing. (Abbreviations: ALI: air-liquid interface).

3D cultures have been found to better reflect *in vivo* gene expression profiles, promoting more correct interpretations of results in comparison to monolayer cultures (Tellez-Gabriel et al., 2018). Therefore, the integration of 3D models in drug screening is essential to validate findings from 2D cultures and accurately assess the effects of anti-cancer drugs (Garnique and Machado-Santelli, 2023). Such approaches have proven to be fruitful for cancer drug screening and are emerging as a pivotal tool in shaping the future of lung cancer research and therapeutic development. Additionally, these culture models have a central role in the principle of the “3Rs” as they support the reduction, refinement and replacement of animal models with systems which could potentially better replicate human diseases and pharmacological responses (Lee et al., 2021). A summary of the main types of 3D cultures used in lung cancer pharmacological research is presented in Figure 3.

The objective of this review is to provide a comprehensive overview of the most recent 3D models and methodologies developed for pharmacological investigations aimed at targeting lung cancer, focusing on advancements made within the past 5 years. Additionally, it seeks to highlight potential research gaps to advance preclinical pharmacology of this highly deadly and heterogenous disease. The common types of 3D cell culture models used to study lung cancer pharmacology are summarized in Table 1.

**TABLE 1:** Common types of 3D cell culture models used to study lung cancer pharmacology.

3D cell culture model	Description	Reference
*Scaffold-based 3D culture models: spheroids and organoids* *Provide a biocompatible supportive structure or matrix in which cells can grow and organize into three-dimensional structures*		
Natural hydrogels	• Derived from natural sources such as collagen, gelatin, or alginate, the latter being derived from seaweed. Simulating the alveolar microenvironment, alginate microbeads coated in human lung fibroblasts have been used to generate small cell lung cancer (SCLC) organoids • Matrigel is a commonly used commercially available hydrogel derived from the EHS mouse sarcoma • Agarose or agar gel microwells or agarose gel coated plates are frequently used as a non-adhesive surface for the generation of spheroids or organoids • om *Engelbreth-Holm-Swarm* (EHS) mouse sarcoma, has been used to generate lung cancer organoids (LCO) • Mimic the native ECM components found in lung tissue, providing a biologically relevant environment which promotes cell-matrix interactions	Sen et al. (2023), Kumari et al. (2020), Gendre et al. (2021), Shabalina et al. (2021), Ilhan-Ayisigi et al. (2020), Luan et al. (2024), Ilhan-Ayisigi et al. (2020), Della Corte et al. (2019), Kim et al. (2019), Shi et al. (2020), Banda et al. (2020), Sulimanov et al. (2023), Sachs et al. (2019), Yokota et al. (2021), Mueggler et al. (2023), Sulimanov et al. (2023), Choi et al. (2021)
*Hydrogels derived from modified natural sources*	• Modified from natural products • Fibrin hydrogels are formed by the polymerization of fibrinogen with thrombin • Chitosan hydrogels are derived from the natural polysaccharide chitin • Provide excellent lung cancer cell adhesion and proliferation but may lack some of the ECM components of purely natural hydrogels	Phogat et al. (2023), Shin et al. (2016)
*Decellularized ECM hydrogels*	• ECM extracted from tissues is processed to remove cellular components, leaving behind the matrix • Retain the native architecture and biochemical composition of the lung tissue ECM.	Shie et al. (2023)
Synthetic hydrogels	• Engineered polymers such as polycaprolactone or polydimethylsiloxane (PDMS) • Offer tunable mechanical and chemical properties, allowing researchers to customise the hydrogel matrix for specific applications • This technology has been described for microfluidic devices including both static and breathing lung-on-a-chip systems	González-Martínez et al. (2020), Tellez-Gabriel et al. (2018), Das et al. (2022)
*Microfabricated hydrogels*	• Hydrogels created using microfabrication techniques such as electrospinning • Enables precise control over the spatial distribution of cells and biochemical cues, enhancing the reproducibility of experiments	González-Martínez et al. (2020)
*Microfluidics with natural hydrogels or decellularized ECM*	• Microfluidic technologies have been combined with natural hydrogels, such as collagen, or dellularized ECM (dECM), to support lung spheroid or organoid growth in a tumour microenvironment (TME)-mimicking micro-architecture	Lee et al. (2019), Knelson et al. (2022), Park et al. (2021), Park et al. (2023)
*Microfluidics with synthetic hydrogels*	• Similarly, microfluidic devices have incorporated synthetic hydrogels such a PDMS with the possibility of hosting lung spheroids or organoids	Tellez-Gabriel et al. (2018), Wan et al. (2023), Jung et al. (2019)
*Scaffold-free 3D culture models* *Allow cells to grow and interact in three dimensions without the use of artificial structures, which can more closely mimic the natural cellular environment found in tissues*		
*Hanging drop technique*	• Tumour cells are allowed to self-assemble within droplets of tissue culture medium hanging from the lid of a dish, and cultured in a high humidity environment	Thirusangu et al. (2022)
*Ultra-low attachment plates*	• Tumour cells are seeded into plates with low attachment coating, and allowed to self-assemble into 3D structures within a tissue culture medium	Thirusangu et al. (2022), Sumkhemthong et al. (2021), Hulo et al. (2024), Huang et al. (2019), Jung et al. (2019), Huang et al. (2020), Seitlinger et al. (2022), Knelson et al. (2022), De Ridder et al. (2022), Wan et al. (2023)
*Magnetic levitation*	• Cells are attached to magnetic nanobeads, and cultured in a plate covered with a strong magnet. The cells levitate within the tissue culture medium, and self-assemble into 3D structures in a simulated low gravity environment	Tepe et al. (2023)
*Angle plate adaptor technology*	• The angle plate adaptor technology is an innovative method designed to facilitate the generation of spheroids. It consists of a specialized device used in conjunction with standard multi-well plates to create a conducive environment for spheroid formation. The key feature of this technology is the angled orientation of the plates, which encourages cells to aggregate into spheroids rather than spreading out as they would in 2D cultures	Vega et al. (2023)
*3D bioprinting* *Various approaches that allow cells to be layered into 3D structures in such a way as to simulate a tumour architecture*		
*Extrusion-based bioprinting*	• Bioink is extruded through a nozzle in a controlled manner to form 3D structures layer by layer • Well-suited for creating larger structures and for incorporating various cell types and hydrogels	Zou et al. (2023), Choi et al. (2023)
*Digital light processing*	• Similar to stereolithography (SLA) but uses a digital light projector to polymerize the resin	Wang M. et al. (2023)
*Microfluidic bioprinting*	• Utilises microfluidic channels to precisely control the flow of bioink containing cells. Enables the creation of microscale structures and facilitates the incorporation of multiple cell types in a controlled manner	Roman et al. (2023)
*Bioprinting with decellularized ECM*	• Bioink is combined with dECM components derived from natural tissues	Shie et al. (2023), Choi et al. (2023)

### 4.1 Lung cancer spheroids

Currently, the term “spheroid” is being used to refer to *in vitro* 3D cultures derived from commercialized cell lines, although a significant overlap between spheroids and organoids exists (Wieleba et al., 2022). The lung cancer spheroid model offers a versatile platform for studying various aspects of the disease. It accurately replicates key features of patient tumours, including cell composition, hypoxic conditions, extracellular matrix deposition, and immunosuppressive microenvironments. This model enables researchers to explore molecular interactions within the tumour microenvironment, identify drug targets, and test potential therapeutics. Additionally, the spheroid model reflects the clonal heterogeneity observed in patient tumours, providing insights into therapy response and drug resistance mechanisms (Rozenberg et al., 2021). Overall, lung cancer spheroids have been instrumental in evaluating the efficacy of anticancer drugs, particularly in assessing drug penetration and resistance within the 3D TME. Moreover, heterotypic spheroids have further enhanced their utility in studying cell interactions and the influence of the TME on therapeutic responses with the aim of developing improved treatment strategies. Lung cancer spheroids are relatively simple and reproducible tumour-mimicking *in vitro* models for screening of novel drugs, including immunotherapies (Boucherit et al., 2020).

#### 4.1.1 Homotypic lung cancer spheroids

Spheroids were generated from A549 cells by seeding in 1.5% agarose-coated 96-well plates for 3 days in supplemented high glucose DMEM. 2D cultures and the 3D spheroids were treated with TTA-A2 (a T-type calcium channel antagonist) alone and in combination with paclitaxel (Kumari et al., 2020). Gendre et al. (2021) derived spheroids from three lung mesothelioma cell lines, H2052/484, H2452 and H2052, which were optimized by seeding at different seeding densities (1,000–100,000 cells/spheroid) using 1.5% agarose-coated 96-well plates. Spheroid growth kinetics (diameter) were monitored via brightfield microscopy over 3 days in culture. Following the selection of the 1,000 cells/spheroid density, each growth kinetics of each model was characterized over 28 days in conjunction with a luminescence-based assay to assess intracellular ATP as a measure of cell viability. A549, H460 and H520 NSCLC cell line spheroids have been developed using the agarose micro-mold technique and characterized for pharmacological research. As a proof-of-concept study, spheroids of these NSCLC cell lines were treated with AZD 2014 (a dual mTOR inhibitor) for up to 6 days using concentrations based on IC_30_ values observed in 2D. AZD2014 was shown to inhibit spheroid growth, shedding light on the increased complexity of these models compared to 2D cultures (Vella et al., 2024).

Spheroids of H1048, H1882, H1876 and DMS53 SCLC cell lines were cultured using the hanging drop method or in ultra-low adhesion (ULA) plates in CSC medium. spheroids were treated with PFK158, a glycolytic inhibitor, and combinations with different chemotherapies to study cytotoxicity (Thirusangu et al., 2022). CSC-enriched spheroids from three lung cancer cell lines, H460, H23 and A549, were formed by culturing in ULA plates for 7 days in glutamine-supplemented DMEM or RPMI medium while lowering the FBS percentage down to 1%. Subsequently, spheroids were dissociated with EDTA and re-seeded into ULA plates for another 14 days. These models were used to study the effects of jorunnamycin A treatment on stem-cell like properties and the resulting chemosensitivity of these spheroids (Sumkhemthong et al., 2021). Vega et al. (2023) developed a novel angle plate adaptor technology for spheroid generation, found to provide similar spheroid formation results to ULA plates. NSCLC spheroids were generated from ten cell lines using this technique and then used to screen 1,280 natural products, resulting in 128 top hits, to potentially identify anti-cancer compounds. A549 transduced with infra-red fluorescent protein (A549-iRFP) have been cultured as spheroids using ULA plates and injected into mice pleura to image tumours *in vivo* for growth analysis. Interestingly, these xenografts progressed through all the four clinical stages of NSCLC (Huang et al., 2019; Huang et al., 2020). In the same year, Li et al. (2020) presented a large-scale CRISPR screening study on lung cancer spheroids in order to identify oncogenes, tumour suppressor genes and novel anti-cancer compounds, while elucidating the increased biomimicry of 3D spheroids over 2D cultures. In another study published in 2020, spheroids generated from several lung cancer cell lines have been used to identify novel potential therapeutic targets with the aim of developing a high-throughput screening methodology. Genome-wide CRISPR screens were conducted on 3D lung spheroids in parallel with lung cells cultured in 2D and mouse xenografts. Comparing the H23 cells cultured in 2D and those cultured as spheroids to the xenografts, the spheroids were found to more accurately identify growth-related cancer vulnerabilities due to the greater ability of spheroids to mimic tumour biology of the mouse xenografts (Han et al., 2020). Furthermore, lung cancer spheroids of a lung adenosquamous carcinoma cell line, H125, were developed using electrospun poly (ε-caprolactone)-based scaffolds of nanometric and micrometric sizes (González-Martínez et al., 2020).

#### 4.1.2 Lung multi-cellular tumour spheroids

Lung multi-cellular tumour spheroids (MCTS) allow for the co-culturing of several cell types to simulate interactions and cross-talks between different cell types and ultimately improve the TME biomimicry of these *in vitro* 3D models.

A NSCLC MCTS model was developed using A549 adenocarcinoma cells co-cultured with BEAS-2B normal human bronchial epithelial cells in agarose gel micro-well molds. These MCTS models were used to study tumour cell migration in different matrices while developing an efficient system for investigation by utilizing a specialized real-time cell analysis system. Through this MCTS model, the early onset of cell migration is exhibited, beginning after only 6 h, highlighting the importance of immediate administration of adjuvant therapy targeting migration. Additionally, this study sheds light on the possibility of the automation of cell migration data analysis (Shabalina et al., 2021). Moreover, A549 spheroids formed on agar micro-well molds were used to study cytotoxicity of propolis and propolis-loaded niosomes (Ilhan-Ayisigi et al., 2020). Spheroids of H358 and A549 cell lines co-cultured with WI-38 human lung fibroblasts were developed using 200 μm agarose gel micro-well molds for drug resistance testing (Luan et al., 2024).

Kaur et al. (2021) developed heterotypic spheroids consisting of 60% HCC827 lung adenocarcinoma cells, 25% human umbilical vein endothelial cells (HUVEC) and 15% mesenchymal stem cells in ULA plates. These cell line-derived lung cancer spheroids together with patient tissues were used to compare treatment responses of four EGFR TKIs with respective 2D cultures. Hulo et al. (2024) generated MCTS models by co-culturing ADCA117, H1975 and H1437 NSCLC cell lines with a fibroblast cell line, human foreskin fibroblast-2 (HFF-2), and magnetically-isolated monocytes from blood donors. Following the generation of MCTS cultures over 3 days using ULA plates, spheroids were treated with combinations of three chemotherapies in clinically similar dosing schemes. These 3D cultures were used to investigate chemotherapy combinations with the aim of identifying enhanced combinational treatments, improving second line therapy and reducing cancer relapse. A 3D lung tumour spheroid platform for oncoimmunology assays was developed using the H1650 adenocarcinoma cell line co-cultured with immune cells and fibroblasts and seeded in ULA plates. These served to provide relevant and easy-to-use models for studying tumour-stroma organization, T cell motility, and immune checkpoint blockade regimens. Additionally, this spheroid model was utilized to assess cytotoxic T lymphocyte (CTL)-mediated killing of tumour cells. By introducing CTLs into the spheroids, the authors were able to monitor target cell-specific killing over time using flow cytometry and live cell imaging. This aspect of the model provides a valuable tool for evaluating the efficacy of CTL-based immunotherapies and investigating mechanisms of resistance to CTL-mediated killing (De Ridder et al., 2022). Using a similar method of generation, A549 MCTS models were developed and treated with chemotherapies between one and 5 days post-seeding. Interestingly, to simulate pharmacokinetics, medium was changed accordingly to mimic the drug blood half-life (Pei et al., 2020).

### 4.2 Lung cancer organoids and tumouroids

Lung organoids are self-organized 3D cultures derived from patient lung tumour tissue, lung stem cells or mesenchymal stromal cells which can replicate tumour or organ tissue by maintaining several characteristics observed *in vivo* (Duzagac et al., 2021; Wieleba et al., 2022; Zhao Z. et al., 2022; Zhu et al., 2023). While 3D models based on cell lines are limited in terms of their biomimicry ability, organoids can replicate tumour or organ structure and heterogeneity, simulating therapeutic responses (Fűr et al., 2024). The first documentation of lung organoids dates back to 1981, while the development of lung cancer organoids (LCOs) for therapeutic screening was first reported in 2019 (Evans and Kaufman, 1981; Kim et al., 2019; Zhu et al., 2023). LCOs are clinically relevant 3D cell cultures, shown to effectively model lung tumours for improved therapeutic investigation and development of precision medicine for specific genetic mutations, potentially minimizing drug failure in the clinical stage and reducing the use of animal models (Lee et al., 2021). Following the first publication of human LCOs, research on LCOs has been drastically increasing since 2016 (Endo et al., 2013; Fűr et al., 2024). The use of LCOs for pharmacological research has gained increasing interest especially over the last 5 years with the number of Pubmed^®^-indexed publications in 2023 exceeding 150 specifically related articles, approximately half of which involved therapeutic studies.

**FIGURE 4:**
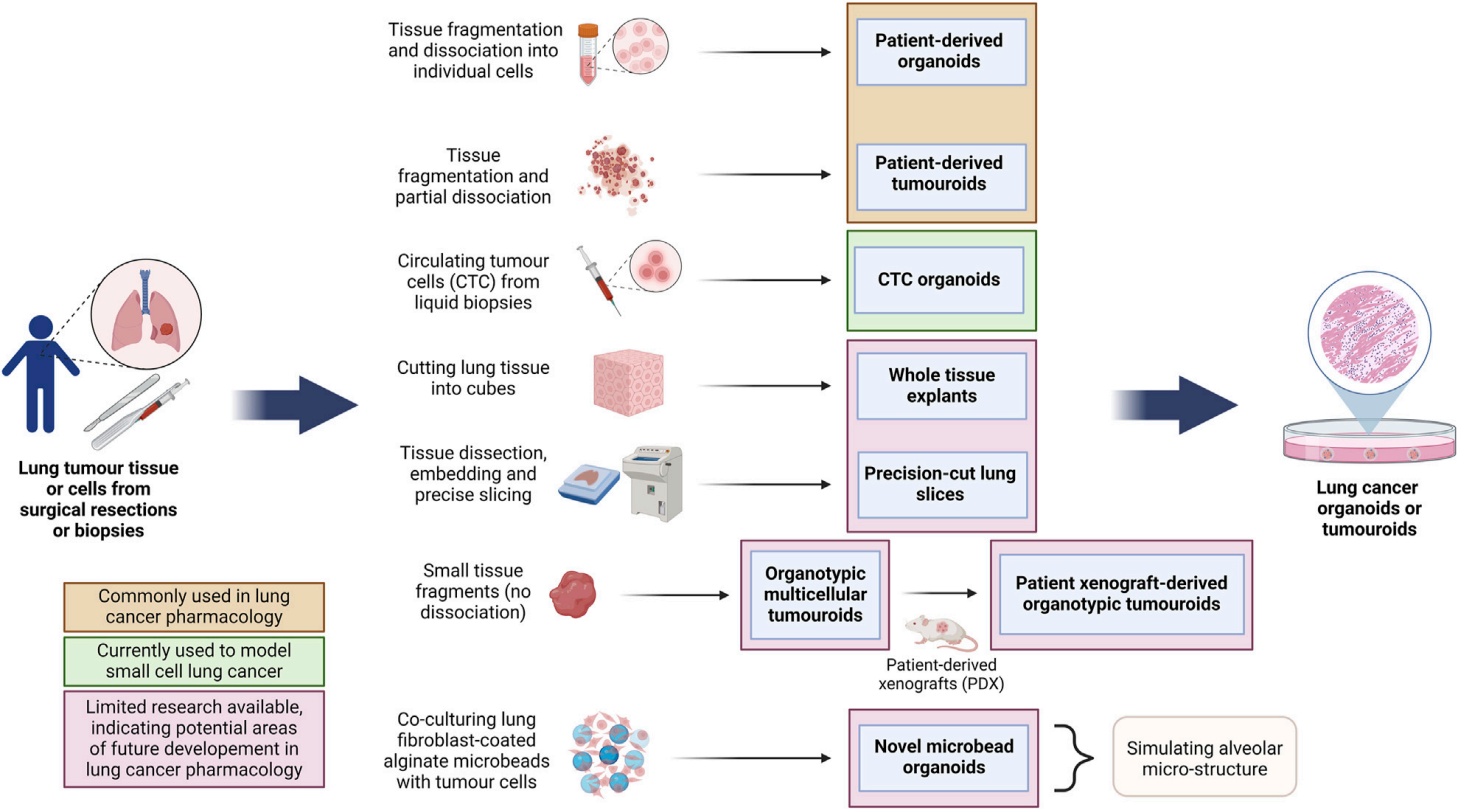
Overview of the methods for the generation of different lung cancer organoids and tumouroids. This diagram illustrates diverse methods employed in the generation of lung cancer organoids and tumouroids, including those derived from patient lung cancer tissue, CTCs from blood biopsies and a novel alveolar-simulating approach. Each method offers unique characteristics which model tumour heterogeneity necessary for accurate drug screening and the development of personalized therapeutics in lung cancer research. Lung cancer PDOs and PDTs have been frequently used in pharmacological studies, while other organoid and tumouroid models represent promising avenues for ongoing advancements in the field. (Abbreviations: CTC: circulating tumour cells, PDO: patient-derived organoids, PDT: patient-derived tumouroids).

When culturing LCOs, each laboratory uses different culture media with different growth factors and inhibitor formulations and varying culture protocols. Currently, it is unclear whether or how these differences affect therapeutic responses *in vitro*. Most LCOs are cultured in a supporting ECM-like material, such as Matrigel or collagen, or otherwise using spinning bioreactors and the air-liquid interface (ALI) method, as discussed in other sections of this review (Huo et al., 2020; Duzagac et al., 2021). Ma et al. (2022) have summarized the latest techniques and growth factors used for culturing LCOs. Currently, partly due to the lack of protocol standardization, LCOs tend to have highly variable success rates ranging from 7% to 87% and risk the overgrowth of normal lung cells. Simpler LCO models are relatively cost-effective, while the more advanced organoid models in combination with animal models, microfluidics and other innovative models tend to be more costly (Kim et al., 2020). Studies involving single-cell RNA sequencing of LCOs have been suggested to better understand the cellular heterogeneity and delve into their tumour-mimicry ability (Lee et al., 2021). A vast range of methodologies have been employed for LCO generation, each one modelling a unique set of characteristics. Figure 4 summarizes different types of lung cancer organoid and tumouroid models with their unique methods of derivation for pharmacological studies.

Although not always clearly differentiated in literature, here we define the most well-understood differences between tumour organoids and tumouroids. The main differences between these two 3D culture models stems from their method of generation: lung cancer organoids are generated from dissociated tumour tissue, while lung tumouroids are formed from fragmented but only partly-dissociated tumour tissue (patient-derived tumouroids), tumour tissue fragments (organotypic multicellular tumouroids, patient xenograft-derived organotypic tumouroids and whole tissue explants) or tumour tissue slices (precision-cut lung slices). This results in different *in vitro* tissue microstructures; while organoids are re-generated from individual cells, the lack of complete tissue dissociation of tumouroids allows the latter to maintain the same complex cellular organization as the *in vivo* tumour from which that specific tumouroid was derived. This preseveration of the *in vivo* microanatomical structure gives tumouroids an advantage in TME studies and personalized medicine research. Table 2 outlines the main differences between these two types of lung cancer 3D models.

**TABLE 2:** Main differences between tumour organoids and tumouroids.

	Organoids	Tumouroids
Derivation	Formed from tumour tissue dissociated into individual cells	Formed from partly-dissociated tumour tissue, small tissue fragments or slices
Architecture	Cellular architecture is restructured after organoid formation *in vitro*	Maintain the complex tumour microenvironment and extracellular matrix, reflecting heterogenous histological and molecular characteristics of the primary tumour
Application	More suitabale for high-throughput drug screening and toxicity testing	More suitable for tumour microenvironment studies and personalized medicine research, allowing for the investigation of patient-specific drug responses

#### 4.2.1 Lung cancer patient-derived organoids

Patient-derived organoids (PDO) are novel *in vitro* models cultured from patient tissues which have been shown to replicate the original tumour biology, including tissue structure, cellular heterogeneity, CSC-driven regeneration and genetic characteristics (Zeng et al., 2023). Numerous studies are showing how lung cancer PDOs are an ideal tool for pre-clinical drug screening and drug resistance studies with the ability of investigating patient-specific responses, paving the way for the discovery of personalized medicines which are critical for the treatment of this highly heterogenous disease (Kim et al., 2019; Wang et al., 2020; Li Y. et al., 2023; Li H. et al., 2023; Liu et al., 2023; Taverna et al., 2024).

A protocol for culturing of lung cancer PDOs was developed following surgical resection or biopsies of lung cancer tissues cultured in Matrigel with minimum basal medium (MBM). These PDOs were cultured for 6 months, over which organoid proliferation was evident from Ki-67 immunolabelling. After characterization, mouse xenografts were generated by the dissociation and transplantation of these organoids. These organoids, both *in vitro* and as xenografts, were then used to screen four anti-cancer drugs (Kim et al., 2019). NSCLC PDO models were generated by dissociation of patient tissue slices and plating tumour cells in Matrigel domes for *in vitro* drug testing (Shi et al., 2020). Choi et al. (2021) developed SCLC PDOs from dissociated tumour tissue biopsies suspended in Matrigel and cultured in supplemented DMEM/F12 which was optimized for long-term SCLC PDO culturing. Moreover, Banda et al. (2020) developed PDOs from homogenized lung adenocarcinoma tissue seeded in Matrigel-coated 24-well plates and characterized for up to 10 days in culture to investigate erlotinib treatment. Lung adenocarcinoma PDO models established by Kim S. K. et al. (2021) were used to predict clinical responses to target therapies and to target specific mutations for the identification of novel molecular targets. Lung tumour tissue was cut and dissociated with collagenase, followed by cell seeding in Matrigel in 48-well plates.

Lung cancer PDO models consisting of tumour cells co-cultured with WI-38 fibroblast cells have been developed with the use of agarose micro-well molds for drug efficacy studies. Co-culturing with fibroblasts was shown to increase resistance to a KRASG12C inhibitor (Luan et al., 2024). Additionally, Sulimanov et al. (2023) cultured PDOs using 96-well plates coated in agarose onto which tumour tissue mixed with a collagen solution was seeded and cultured in supplemented DMEM/F12. Using this culture model, the authors equated a mathematical model describing the interactions of different cell types in NSCLC tumours in order to predict cellular sub-population alterations over 7 days with the aim of translation to the clinic. Yokota et al. (2021) developed PDOs for therapeutic screening from lung adenocarcinoma, lung adenocarcinoma lymph node metastases and EGFR-resistant lung cancer malignant pleural effusion cells using basal membrane extract. The cell culture medium was optimized for cellular purity and long-term expansion (up to 13 months). In parallel, PDOs were cultured from adjacent normal lung tissue (Sachs et al., 2019).

Of interest, lung cancer PDOs in ALI conditions were used to model the lung immune checkpoint blockade for immunotherapy studies. These PDOs were shown to maintain immune cells and their receptors *in vitro* (Neal et al., 2018). Otherwise, the culturing of lung cancer spheroids or organoids in ALI conditions has been rarely reported.

#### 4.2.2 Circulating tumour cell lung cancer organoids

The concept of using circulating tumour cells (CTCs) for LCO generation has been recently proposed as tools for disease modelling, drug screening and identification of personalized medicine (Yang et al., 2019). Considering the relative difficulty of obtaining SCLC patient tissues, and the ease of CTC collection through liquid biopsies and their abundance in SCLC patients (especially at the advanced stage), this LCO model may pave the way for novel pharmacological research employing SCLC organoids (Huo et al., 2020; Fűr et al., 2024). SCLC CTC LCOs cultured in a polydimethylsiloxane (PDMS)-based microfluidic device in co-culture with CAFs and immune cells have been shown to closely simulate genetic mutations of the primary tumour (Zhang et al., 2014; Tellez-Gabriel et al., 2018). Hamilton and Rath (2019) established nine SCLC CTC cell lines which spontaneously formed LCOs in culture. The authors discuss the role of modelling aggressive metastatic tumours from these cell lines for metastasis and chemoresistance studies. However, *in vitro* models of SCLC and CTC LCOs require further progress.

#### 4.2.3 A novel organoid formation technique to simulate the alveolar micro-structure

Unique organoid models have been developed by making use of alginate microbeads coated in primary human lung fibroblasts and co-cultured with SCLC cell lines H526, H1963 and H82, forming organoids after 7 days. These were used to study the therapeutic effects, including relapse, of the chemotherapeutic compounds cisplatin and etoposide. Characterization revealed high similarity between these organoids and patient tumours, and were shown to better simulate *in vivo* SCLC tumours (Sen et al., 2023).

#### 4.2.4 Patient-derived tumouroids

Patient-derived (or tissue-derived) tumouroids (or tumourospheres) (PDT) are generally formed by culturing of fragmented and partly-dissociated tumour tissue (Daunys et al., 2021).

Knelson et al. (2022) generated lung cancer PDT models to study the effects of an agonist of the activation of the stimulator of interferon genes (STING) pathway with the aim of activating innate anti-tumour immunity. Fresh tumour specimens were minced on ice and resuspended in supplemented DMEM, cultured in ULA plates or embedded in collagen (Jenkins et al., 2018). Additionally, PDTs were loaded onto a microfluidic device to generate biomimicry tumour-vascular models.

Other *ex vivo* spheroid generation protocols involved the processing of NSCLC tissue samples by mincing and enzymatic digestion followed by seeding in Matrigel or in 2.5% agarose gel 256-microwells. PDTs grown in Matrigel were used to study the effects of anti-PD-L1 immunotherapies (atezolizumab and avelumab) and an MEK targeted therapy (selumetinib), while those in agarose microwells were used for studying chemotherapeutic combinations 3 days after seeding (Della Corte et al., 2019; Mueggler et al., 2023). Lung adenocarcinoma primary tissues processed with or without enzymatic digestion were used to develop spheroids by seeding in 1.5% agarose-coated 96-well plates, cultured in complete RPMI. These spheroids were optimized as previously described in the study by Gendre et al. (2021). Di Liello et al. (2019), report the similarity of treatment responses in NSCLC patients and their respective *ex vivo* PDT models. Lung cancer tissue was dissected, enzymatically digested and seeded in Matrigel. PDTs were able to replicate chemotherapy and immunotherapy responses, while opening up on the possibility to predict patient outcomes and personalized medicine. Seitlinger et al. (2022) developed an *in vitro* vascularized NSCLC 3D model by cutting and enzymatically digesting patient-derived lung tumour tissue and co-culturing these tumour cells with fibroblasts and endothelial cells in ULA plates. Combined with a pre-vascularized fibrin matrix, the authors note the potential that these PDT models have for incorporation into a microfluidic device for improved anti-cancer drug screening (Lê et al., 2022).

#### 4.2.5 Patient xenograft-derived organotypic tumouroids

Organotypic multicellular spheroids (OMS) are formed by culturing small fragments of tumour tissue without prior dissociation, favouring the maintenance of the *in vivo* tumour architecture. Ivanova et al. (2020) report the establishment of patient xenograft-derived organotypic tumouroids (PXDOT) from patient-derived exon 19 and exon 20 *HER2*-mutant NSCLC xenografts (PDX). These PDX tumours were processed and cultured in ULA plates for spheroid formation, followed by transferring spheroids to microfluidic devices and exposed to different drugs and drug combinations (Jenkins et al., 2018).

#### 4.2.6 Lung cancer whole tissue explants

The lung whole tissue explants (WTE) method involves cutting lung tissue into cubes, providing a three-dimensional perspective similar to PCLS. While it may not fully retain the airway’s 3D structure, it has proven effective in studying various stimuli responses in lung tumours. Adapted WTE models were used to investigate human NSCLC chemotherapy and targeted therapy responses, demonstrating the model’s reliability and reproducibility (Karekla et al., 2017). Although WTE requires less preparation than PCLS, comparing different experimental conditions can be challenging due to potential variations in cell type ratios (Pomerenke, 2021). Evans et al. (2021) generated *ex vivo* models from 1 mm^3^ NSCLC WTE to demonstrate the advantage of using this model in therapeutic research due to their ability in sustaining an immunosuppressive environment which can mimic the TME better than spheroid co-culture models (Evans et al., 2021). Pharmacological research involving lung cancer WTE is evidently still very lacking.

#### 4.2.7 Lung cancer precision-cut lung slices

Precision-cut lung slices (PCLS) are thin sections of lung tissue that are prepared using precise cutting techniques to maintain the structural and functional integrity of the tissue. These slices typically range from a few hundred micrometres to a few millimetres in thickness and are used as *ex vivo* models to study lung physiology and disease, including lung cancer pharmacology. Following tissue dissection, embedding in low-melting agarose and precise slicing with specialized equipment, such as a vibratome, these tissues are prepared for culturing. Human PCLS models from tumour samples could be valuable in studying tissue responses in preclinical models of anti-cancer therapy. This organotypic model can be used to simultaneously study various aspects of lung function, including airway reactivity, immune responses, drug metabolism, and toxicology (Alsafadi et al., 2020; Närhi et al., 2018).

Overall, PCLS provide a robust and reproducible model to study the effects of various stimuli in a controlled *ex vivo* environment. They offer flexibility in testing multiple conditions from the same donor and can be maintained viable for extended periods *in vitro*. Efforts are ongoing to validate PCLS as a model for drug toxicity testing in preclinical settings (Pomerenke, 2021). Interestingly, NSCLC PCLS *ex vivo* cultures were found to retain cellular organisation for up to 12 days in culture (Junk et al., 2021). However, Preuß et al. (2022), caution about the changes in cell populations over long-term PCSL culture. Currently, no studies have been published using PCLS models for lung cancer disease progression modelling, and it has been suggested that such studies take into consideration the cutting damage which progresses during cultivation (Alsafadi et al., 2020; Preuß et al., 2022). Alsafadi et al. (2020) discuss the possible applications of PCLS in lung cancer pharmacology, including novel drug discovery and delivery, cancer cell resistance and personalized medicine. However, pharmacological research using models of PCLS from human lung cancers is still lacking and requires a better understanding in order to take advantage of its TME-replicating potential in preclinical lung cancer pharmacology.

### 4.3 Microfluidics

Microfluidic devices comprise a broad category of micro-fabricated devices designed for 3D cell culture technology. They are commonly referred to as “organ-on-a-chip” or “lab-on-a-chip” systems, and consist of tiny channels and chambers etched into materials such as PDMS or plastics. Cells are cultured in the chamber areas while fluids are pumped at precisely controlled rates through the channels, simulating a circulatory system. The lung-on-a-chip model, conceived over 15 years ago (Huh et al., 2010; Huh, 2015; Francis et al., 2022) has gone through several iterations of improvement and presents a formidable approach towards designing a TME over which a liquid containing nutrients, oxygen, drugs, assay compounds and even other cells, can flow at finely controlled rates. Besides addressing up-scalability and throughput issues, lung-on-a-chip models also allow for the study of the drug effects on lung cancer metastasis.

The mechanical breathing motion occuring within the lungs is thought to alter cellular behaviour leading to changes related to several hallmarks of lung cancer, including progression, angiogenesis and metastasis. Breathing lungs-on-a-chip have been designed to model lung disease and progression and to study drug responses in lung pathologies including lung cancer, using systems design to simulate the biomechanical TME of breathing lungs. This technology involves the use flexible material combined with cyclic motion usually by applying negative pressure via vacuum chambers, simulating the physiological movement of lung alveoli. As in other microfluidic devices, these lung cancer models have been co-cultured with several other cell types such as endothelial cells and fibroblasts (Sontheimer-Phelps et al., 2019; Barros et al., 2021; Das et al., 2022). The growing interest in these multi-dynamic models has the potential to significantly enhance cell culture biomimicry, driving advancements in drug development and personalized medicine research for lung cancer (Shrestha et al., 2020; Francis et al., 2022).

A multi-organ microarray containing three layers of chambers that mimicked the invasive microenvironment of lung cancer, has been used to assess the mechanism of EMT in lung cancer cells which invaded distant tissues and organs, such as the brain, bone, and liver (Xu et al., 2016). This body-on-a-chip multi-organ model was further developed by the same research team, in 2020 (Xu et al., 2020), to construct a microarray model of lung cancer brain metastases to specifically investigate the mechanisms underlying metastatic resistance to chemotherapeutic drugs. One year later, Zheng and co-workers reported a microarray lung cancer model designed to study liver metastasis in hypoxic conditions, focusing on the therapeutic effects of HIF-1 inhibitors on invasion (Zheng et al., 2021; Zhu et al., 2023). More recently, more complex body-on-a-chip models incorporating a broader range of body tissues, and novel micro-biosensors have been developed for lung cancer studies (Jalili-Firoozinezhad et al., 2021; Ding et al., 2021), and the data outputs suggests such models to be a formidable evolving technology with strong applications both in industrial R&D as well as academic research environments.

Termed as the quantum leap in cancer research, efforts are being made to integrate lung cancer spheroids and organoids into microfluidic set-ups (Duzagac et al., 2021; Wu Y. et al., 2021; Carvalho et al., 2022). A549 spheroids were developed using purposely designed microfluidic devices which allow for the co-culturing of HUVEC cells in a spatial arrangement over collagen-embedded spheroids. Hence, the microfluidic channel through which medium passes is lined by a layer of endothelial cells forming biomimicry blood vessels proximal to the A549 spheroids embedded in an ECM-like material. This serves as an ideal model for studying interactions between tumour and endothelial cells, angiogenesis and invasion and how these are affected by drug treatments (Lee et al., 2019). Another drug-screening model was developed by generating triple co-culture spheroids consisting of A549 cells, human lung fibroblasts and HUVECs followed by their incorporation into a microfluidic device with a pro-angiogenic porcine lung decellularized ECM-based hydrogel and a vascularized system (Park et al., 2021; Park et al., 2023). Wan et al. (2023) describe a protocol to enhance vascularization in lung cancer spheroids-on-a-chip by introducing lung fibroblasts after spheroid formation and demonstrate the improved immune responses using this novel model. In this study, spheroids of a mesenchymal derivative of the H69 SCLC cell line (H69M) were co-cultured with fibroblasts, endothelial cells and pericytes in ULA plates and transferred to a PDMS-based microfluidic device having a central gel channel surrounded by two media channels simulating blood vessels. In 2019, a PDMS-based microfluidic device was designed to replicate lung micro-physiology and support the formation of low size variation lung cancer PDOs for drug testing using cells seeded in Matrigel. This is reported to be the first SCLC PDO model for pharmacological research (Jung et al., 2019).

Recent developments in organoid-on-a-chip models have managed to recapitulate lung tumour vascularization, enhancing drug delivery and drug screening research (Kim S. K. et al., 2021; Wu Y. et al., 2021). Organoids-on-a-chip enable high-throughput drug screening, automatic treatment schedules and allow simultaneous monitoring of drug reactivity parameters while highly simulating the TME (Zeng et al., 2023).

### 4.4 Bioprinting

Cell bioprinting represents an emerging transformative approach in lung cancer research, offering the potential to recreate complex tissue architectures *in vitro* with a high degree of precision. This innovative technology has the capability to fabricate 3D structures by sequential deposition of bioinks - materials that contain living cells and biomaterials - therefore forming functional living tissues.

In lung cancer research, bioprinting is showing increasing applications, such as the generation of tumour-stroma constructs by co-printing cancer cells with stromal elements, such as fibroblasts and ECM components. This provides a dynamic system to investigate the interactions between cancer cells and the ECM, which are crucial for understanding cancer progression and the impact of the TME (Herreros-Pomares et al., 2021; Falcones et al., 2021). Bioprinted lung models can be leveraged for drug screening and toxicity testing and can potentially contribute to personalized medicine in lung cancer treatment by enabling the production of patient-specific tumour models using the patient’s own cells.

Despite the promising applications, some challenges remain, such as replicating the full complexity of lung tissue, including the vascularization needed for nutrient and oxygen supply to the deeper cell layers, as well as ensuring long-term viability and functionality of the bioprinted constructs. The high financial investments and running costs required for a bioprinting facility also need to be considered and justified, in view of other 3D lung models which are cheaper to generate.

### 4.5 Decellularized lung matrices

3D lung cancer models supported by decellularized ECM (dECM) mimic important elements of the ECM and cell-ECM interactions. The unique molecular composition and structure of these bioactive scaffolds can significantly improve pre-clinical pharmacological research by taking into account tumour physiological parameters which are frequently overlooked (Hoshiba, 2019; Ferreira et al., 2020; Ferreira et al., 2021; Marques-Magalhães et al., 2022).

Shie et al. (2023) aimed to develop a biomimetic spheroid model derived from HCC827 lung adenocarcinoma cells to study drug responses to several EGFR TKIs. These lung cancer cells were co-cultured with THP-1 (leukaemia monocytes), human pulmonary fibroblasts and HUVECs. Cells were mixed with dECM extracted from bovine lungs and seeded as droplets in 96-well plates via an automated printing needle (5,000 cells/spheroid). A solution of dECM without cells was added to cover the droplets and once gelation was ready, culture medium with HUVECs were added. After 5 days, diameters of most of the spheroids ranged between 300 and 400 μm, allowing for cell layer organization and necrotic core formation. To validate these models for pharmacological research, cytotoxicity with several EGFR TKIs was evaluated in dECM-embedded spheroids of HCC827, H1650, H3255, and GR10 lung cancer cell lines. Two and 5 days post-treatment, cell viability was analysed by live/dead staining using Calcein AM/Ethidium homodimer-1 (EthD-1) and confocal microscopy. In addition, HCC827 models were treated with chemotherapeutic combinations to analyse drug responses (Shie et al., 2023). As previously discussed, the use of lung dECM has been employed in a spheroid-on-a-chip microfluidic device to study angiogenesis (Park et al., 2021; Park et al., 2023).

The ability of such bioactive scaffolds to recapitulate complex TME interactions, brings to light the possibility of utilizing lung tumour dECM based on the stage and location of the lung cancer model being investigated, or using dECM surrounding the same tumour tissue used in culture. Further exploration into the construction of 3D lung cancer models based on lung tumour dECM is highly encouraged due to its potential to significantly improve the advancement of anti-cancer strategies and precision medicine.

### 4.6 Air-liquid interface

**FIGURE 5:**
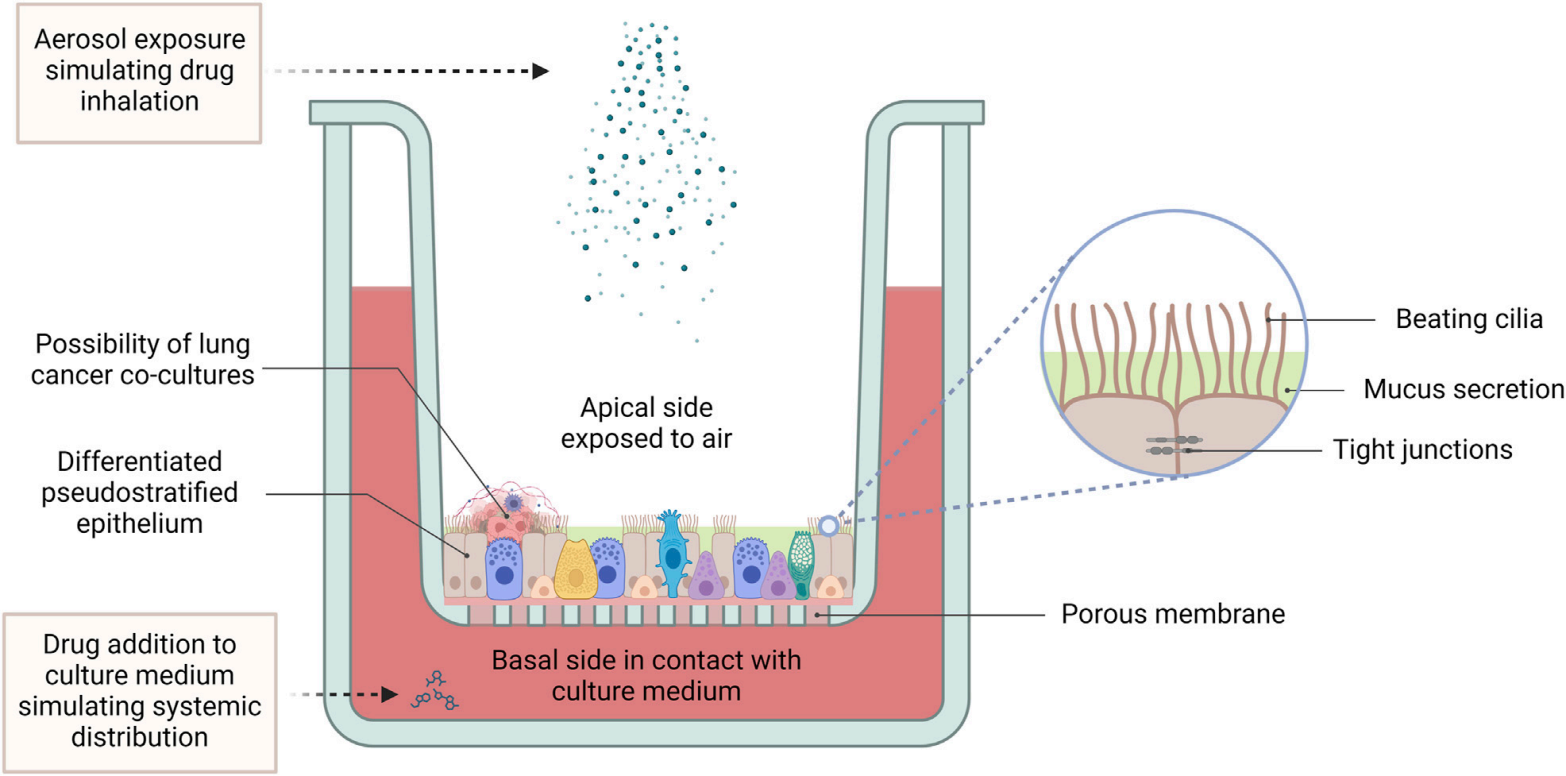
The air-liquid interface culture set-up. In ALI conditions, primary lung cells differentiate into a pseudostratified pulmonary epithelium, featuring beating cilia, mucus production, and tight junctions between epithelial cells. While harbouring the possibility of co-culturing with lung tumour cells, this advanced 3D model provides a physiologically relevant platform for pharmacological studies, facilitating the investigation of lung cancer pathogenesis, drug responses and therapeutic efficacy. ALI cultures allow for application of treatment to the apical side using aerosolized drugs to simulate inhalation therapy, while treatment applied to the culture medium mimics systemic drug distribution. (Abbreviations: ALI: air-liquid interface).

Air-liquid interface (ALI) culturing provides a method of culturing airway epithelial cells, in such a way that they are exposed to the air while still absorbing nutrients from a tissue culture medium. Such cultures are prepared by seeding and culturing airway epithelial cells onto micro-well inserts composed of a collagen-coated polyethylene terephthalate (PET) porous membrane. The apical surface of the cells is exposed to the air, while they absorb nutrients from the tissue culture medium, through the basal side which is adhered to the insert. This mode of culture enables the development of a ciliated morphology and tight junctions similar to that observed *in vivo*, giving an overall pseudostratified epithelium appearance and better recapitulation of the structural architecture and differentiated functions of the respiratory epithelium (Figure 5). This leads to a more accurate representation of cellular and molecular changes associated with lung cancer (Agraval et al., 2022).

Indeed, Geles and co-workers demonstrated that NSCLC primary cells cultured using the ALI model allows for the examination of gene networks and differentiation potential in a 3D growth environment that mimics the *in vivo* lung environment. Using this model, the researchers also showed that poorly differentiated cells overexpress RNA processing factors and exhibit a distinct profile of alternatively spliced mRNAs, compared to well differentiated NSCLC cells, suggesting that alternative post-transcriptional mechanisms may be occurring in poorly differentiated lung cancer cells (Geles et al., 2016). More recently, Zhao et al. (2024) used an ALI model generated using tumour tissues from patients with stage IA NSCLC and used it to show that in the presence of low concentrations of interferon-γ, upregulation of the *IDO1* gene occurred. This enhanced the viability of cancer cells, while inhibiting the viability of T cells and NK cells, therefore establishing an immune microenvironment conducive to tumour progression.

Another application for which lung cancer ALIs have been used, was to simulate lung inhalation by spraying gene drugs onto the ALI apical surface, in order to assess the effectiveness of administering gene therapy via the inhalation route. This method allowed for the assessment of the effects of inhalable gene transfection on cell proliferation and gene expression under conditions that are more representative of the actual lung environment (Ichikawa et al., 2022).

## 5 Pharmacological responses in 3D vs*.* 2D models of lung cancer

### 5.1 Drug responses in spheroids

HCC827 spheroids treated with erlotinib, afatinib or lapatinib for 96 h exhibited higher sensitivity than the 2D cultures. Furthermore, spheroids were more sensitive to osimertinib than the respective 2D cultures (Kaur et al., 2021). A comparative study using A549 2D cultures and 3D spheroids treated with TTA-A2 monotherapy or in combination with paclitaxel was conducted by Kumari and colleagues. IC_50_ was reached at 50 nM in 2D A549 cultures, while A549 spheroids exhibited an IC_50_ of 100 nM. The combinational therapy seemed to be more effectively recapitulated in spheroids, rather than the 2D cultures (Kumari et al., 2020). Other A549 spheroids generated on agar gel micro-wells were treated with propolis for three and 7 days. Fluorescence staining and imaging showed minor cytotoxicity and a slight increase in cell scatter with propolis treatments (Ilhan-Ayisigi et al., 2020). Spheroids generated from wild-type HCC78 cell line and three HCC78 *ROS1*-mutants, exhibited different responses to five TKIs: lorlatinib, entrectinib, crizotinib, repotrectinib and ceritinib. Interestingly, these differences in therapeutic responses were not observed in treated 2D cultures (Terrones et al., 2024). Three days post-seeding H2052/484 lung mesothelioma cells (1,000 cells/spheroid), spheroids formed were treated with cisplatin (50, 100, or 200 μM) or cisplatin/pemetrexed combination (50/200 μM, 100/400 μM or 200/800 μM) for 3 h, then washed and cultured for 17 days. IC_50_ was estimated by an ATP-based luminescent assay 24 h post-treatment: cisplatin = 1.21 μM; cisplatin/pemetrexed = 3.18/12.72 μM (Gendre et al., 2021).

H460, H23 and A549 spheroids generated in ULA plates exhibited lower IC_50_ values compared to normal bronchial cell line, BEAS-2B, spheroids upon treatment with non-toxic concentrations of jorunnamycin A (0.05–0.5 µM) over 7 days. Cytotoxicity was measured using annexin V-fluorescein isothiocyanate (FITC)/propidium iodide (PI) flow cytometry analysis. Spheroid sizes with treatments were analysed and were reduced at a time and dose-dependent manner. Additionally, jorunnamycin A (0.5 µM) was found to sensitize H460 spheroids to cisplatin treatment (25 µM) (Sumkhemthong et al., 2021). ULA-generated A549 MCTS cultures exhibited higher IC_50_ values in the 3D compared to 2D cultures when treated with cisplatin (20.71 μM vs. 9.73 μM), carboplatin (188.90 μM vs. 131.80 μM), doxorubicin (6.57 μM vs. 0.61 μM) and gemcitabine (>250 μM vs. 0.027 μM) for 72 h (Pei et al., 2020).

SCLC spheroids generated from H1048, H1882, H1876 and DMS53 cells were treated with PFK158 (1, 2.5 and 5 μM) 7 days post-seeding (Mondal et al., 2019). After 24 h post-treatment, an overall decrease in IC_50_ values was observed in treated spheroids compared to the 2D cultures of the same cell lines. Numbers and sizes of spheroids decreased with treatment, while invasion was inhibited with 5 μM PFK158. In combination studies, PFK158 was found to increase the sensitivity to several conventional chemotherapeutic drugs. Cell viability was assessed via trypan blue dye exclusion assay and spheroid diameters were monitored via inverted microscopy (Thirusangu et al., 2022).

### 5.2 Drug responses in organoids and tumouroids

Lung cancer PDOs developed by Kim et al. (2019) were used for therapeutic screening of docetaxel (IC_50_ = 0.08 μM), olaparib (IC_50_ = 69 μM), erlotinib (IC_50_ > 100 μM) and crizotinib (IC_50_ = 3 μM), obtaining IC_50_ values via a luminescence-based ATP assay after 6 days of treatment. Immunoblotting of lysed PDOs with EGFR and c-MET-related proteins showed changes in expression with erlotinib (1 μM) and crizotinib (1 μM) after 24 h, 48 h and 72 h. In another study, NSCLC PDO models were used to study the targeted therapies trametinib and selumetinib (MEK inhibitors), afatinib (EGFR inhibitor), BKM120 (PI3K inhibitor) and BGJ398 (FGFR inhibitor) targeted therapies at 0.01–10 μM concentrations for 96 h. Cell viability was measured via ATP assays. IC_50_ values obtained were dependent on the types of mutations present in each PDO model. However, combinations of BGJ398 with BKM120 exhibited some synergistic effects, while BGJ398 with trametinib exhibited strong synergism (Shi et al., 2020). Lung adenocarcinoma PDO models were seeded with four concentrations of erlotinib (0.01, 0.1, 1, or 10 μM) for 2 weeks, followed by treatment with 10 μM for 24 weeks (with several passages and re-platings), always changing the medium every two to 3 days. Confocal and brightfield images were collected over the course of treatment to study morphological characteristics. Moreover, DNA was isolated from the organoids followed by multiplex PCR for mutational analyses (Banda et al., 2020). As reported by Kaur et al. (2021), a PDO model treated with lapatinib exhibited a lower IC_50_ value than monolayers. In other models, therapy responses of 3D lung adenocarcinoma PDOs and their 2D counterparts were compared after three-day treatments resulting in different responses with different treatments and according to different tumour mutations present in the tumour tissue used. PDOs were treated for up to 15 days and cell viability was measured using an ATP-based luminescent assay (Kim S. Y. et al., 2021). Mueggler et al. (2023) used the agarose micro-well-generated lung adenoid cystic carcinoma PDTs to study therapeutic responses of seven concentrations of cisplatin/etoposide (6.25/4.2–400/270 μM) and cisplatin/paclitaxel (6.25/5–400/320 μM) combinations for 18 days. An ATP-based assay was used to study cell viability, while PDT diameters were monitored via imaging. IC_50_ was reached at 84/57 µM for cisplatin/etoposide and 103/82 µM for cisplatin/paclitaxel and PDT diameters exhibited a dose-dependent decrease in size.

SCLC PDO models were used for studying the pharmacological effects of cisplatin and etoposide treatment was studied using these SCLC PDO models. 200 single cells were mixed with Matrigel and suspended into 96-well plates. After 2 days, PDOs were treated with cisplatin and etoposide at 0.01, 0.1, 1, 2, 5, or 10 μM for 8 days. Cell viability was assessed by measuring ATP content, attaining IC_50_ values at 12.39 μM and 0.5382 μM, respectively. Tumour recurrence was studied by withdrawing treatment after 10 days and replacing with fresh medium. SCLC PDOs were able to re-grow to their original size after 2 weeks of 10 μM cisplatin treatment. Cells within the organoids were able to resist 50 μM cisplatin treatment and were shown to grow after 4 weeks of cisplatin withdrawal (Choi et al., 2021). Other SCLC PDOs embedded in a microfluidic device (diameter ˂ 200 μm) were used to test responses to cisplatin (20–80 μM) and etoposide (10–40 μM) over 72 h of treatment. This induced apoptosis which was assessed by staining with Annexin V-FITC/PI and caspase-3 with 4′,6-diamidino-2-phenylindole (DAPI) counterstaining. Cleaved caspase-3 anti-body was used to assess cellular apoptosis with immunofluorescence microscopy. Interestingly, PDO centres were found to survive even at the high concentrations, regardless of the small sizes of the organoids (Jung et al., 2019).

Combinational therapy of MAPK (NVP-BEZ235/dactolisib; 0.5 and 1 μM), PI3K/mTOR (AZD6244/selumetinib; 0.5 μM) and the SRC (saracatinib; 1 μM) inhibitors has been conducted on lung cancer PCLS models. Following 24-h treatments, alterations in signalling pathways and necrotic cell populations were assessed by immunohistochemistry (IHC) and biomarker analysis (Alsafadi et al., 2020; Närhi et al., 2018).

*HER2*-mutant microfluidics-embedded PXDOT models were used to study therapeutic responses to gefitinib, afatinib and neratinib (all at 0.5 μM) over 96 h using dual immunofluorescent staining to identify live/dead tumour cells. Combinations of neratinib with AZD8055 (1 μM) and trastuzumab (10 μg/mL) showed that the combination of AZD8055 and trastuzumab was ineffective at reducing tumour cell viability. However, trastuzumab and neratinib led to 50% loss of viability, while the combination of AZD8055 (35% decrease) and neratinib (20% decrease) exhibited enhanced therapeutic effects compared to the individual treatments (60% decrease).

### 5.3 Drug responses in static versus breathing lungs-on-a-chip

Hassell et al. (2018) developed a breathing alveolus lung-on-a-chip involving primary normal lung cells co-cultured with H1975 adenocarcinoma NSCLC cells which were GFP-transfected for microscopic visualisation. When comparing static versus breathing chips, cancer cell growth and invasion decreased significantly with biomimetic breathing. In this study, the presence of cyclic breathing was shown to increase resistance to EGFR therapies, exhibiting almost complete resistance to both 100 and 1,000 nM of rocicletinib over 6 days of treatment. This study suggests that TKI drug responses of such biomechanical orthotopic breathing lung-on-a-chip models parallel drug responses observed *in vivo*.

## 6 Common pharmacological assays applicable to lung cancer 3D models

Pharmacological research using 3D cell culture models requires the assessment of a spectrum of parameters arising from the increased complexity of these tumour models. The common laboratory-based assays used for pharmacological studies are described below and summarized in Table 3.

**TABLE 3:** Common laboratory-based assays used for pharmacological studies in lung cancer 3D cell culture models.

Applications to lung cancer pharmacology	Types of assays	Description	Reference
Cell viability	Cell staining for fluorescent imaging and/or flow cytometry	*Examples of commonly used live/dead cell stains:* Live cell stains: Calcein acetomethoxy (AM), fluorescein diacetate (FDA) and acridine orange (AO) Dead cell stains: Propidium iodide (PI) and Ethidium homodimer-1 (EthD-1) Trypan blue extrusion and manual counting has also been used for live/dead analyses Other commercially available live/dead stains have been used	Ivanova et al. (2020), Lee et al. (2019), Jung et al. (2019), Evans et al. (2021), Thirusangu et al. (2022), Kumari et al. (2020), De Ridder et al. (2022), Ilhan-Ayisigi et al. (2020)
	Colorimetric Assays	Common dyes or commercially available assays used to study drug-induced cytotoxicity in lung cancer 3D cell cultures	
	ATP-based luminescence assay	The assay reagent contains a luciferase enzyme and its substrate, luciferin, along with other components that facilitate penetration through 3D cell layers of spheroids and organoids, inducing cell lysis and driving the release of intracellular ATP. The released ATP reacts with luciferin in the presence of luciferase, producing a luminescent signal. The intensity of the luminescence is directly proportional to the amount of ATP.	Huang et al. (2020), Shi et al. (2020), Thirusangu et al. (2022), Kim et al. (2019), Kim S. Y. et al. (2021), Gendre et al. (2021)
	Fluorescent assay based on cell membrane rupture	The assay reagent contains a green fluorescent dye that is impermeant to live cells but can penetrate compromised cell membranes of dead cells within spheroids and organoids. Binding of the dye to the DNA of dead cells, emits a fluorescent signal which can be quantified by spectrophotometry and visualised by fluorescence microscopy. This assay supports multiplexing with the previously mentioned ATP-based luminescent assay to obtain complementary data on cell viability and cytotoxicity	Koudan et al. (2021), Appleton et al. (2021), Barbosa et al. (2021)
	Lactate Dehydrogenase (LDH) release assay	The enzyme LDH is present in the cytoplasm of cells. When the plasma membrane is damaged, LDH is released into the surrounding culture medium and can be detected as an indicator of cytotoxicity. LDH catalyses the conversion of lactate to pyruvate while reducing NAD+ to NADH, which in turn interacts with a tetrazolium salt in the presence of an electron acceptor, converting it to a coloured formazan product	Thirusangu et al. (2022), Barbosa et al. (2021)
	MTT assay	A measure of cell metabolic activity, which is often interpreted as a measure of cell viability, proliferation, and cytotoxicity. The assay relies on the ability of living cells to convert the yellow tetrazolium salt 3-(4,5-Dimethylthiazol-2-yl)-2,5-Diphenyltetrazolium Bromide (MTT) into a purple formazan product, which can be quantitatively measured	Daunys et al. (2021), Della Corte et al. (2019), Di Liello et al. (2019)
	Resazurin-based assay	Resazurin is a non-toxic, cell-permeable dye. In metabolically active cells, resazurin (blue) is reduced by mitochondrial enzymes to resorufin (pink), a highly fluorescent compound. The reduction process reflects cellular metabolic activity, therefore providing a measure of cell viability	Shie et al. (2023)
Apoptosis and cell cycle analysis	Cell staining and flow cytometry	Most common staining is Annexin V-Fluorescein isothiocyanate (FITC) (apoptotic marker) and counterstaining dead cells with PI. 3D cultures are dissociated using gentle enzymatic reagents to preserve the cell surface markers. Cells are then stained with the appropriate dyes and read by flow cytometry	Jung et al. (2019), Sumkhemthong et al. (2021)
	Cell staining and immunofluorescence microscopy	Cleaved caspase-3 antibody has been used for apoptotic visualisation in lung cancer 3D models. 4',6-diamidino-2-phenylindole (DAPI) is commonly used nuclear counterstain	Jung et al. (2019)
Morphological and mechanical analysis	Brightfield microscopy	Used to image spheroids or organoids in 2D followed by measuring of several morphological characteristics by specialized software	Jung et al. (2019), Gendre et al. (2021), Banda et al. (2020), Sumkhemthong et al. (2021), Ilhan-Ayisigi et al. (2020)
	Confocal microscopy	Combined with fluorescent staining or immunostaining, allows for the 3D visualisation of specific cells or markers on the surface and through all cell layers	Shie et al. (2023), Banda et al. (2020), Mueggler et al. (2023)
	Atomic force microscopy	Used to study spheroid or organoid stiffness. Stiffness is a crucial parameter that influences cellular behaviour, including cell proliferation, differentiation, migration and apoptosis	Sen et al. (2023)
	Immunohistochemistry (IHC)	Following cryosectioning or paraffin-embedding procedures, slices of lung cancer 3D cultures can be stained accordingly. A commonly used IHC stain is the Haematoxylin and Eosin (H&E) stain to study the micro-tissue architecture within the models	Kumari et al. (2020), Charlet-Faure et al. (2023), Yoshimoto et al. (2023), Mueggler et al. (2023), Kim S. Y. et al. (2021)
	Immunofluorescence	Immunostaining of several proteins commonly used to identify specific cellular populations or characteristics (e.g., cell proliferation and metastatic markers) Many commercial dyes are available	Daunys et al. (2021), Ivanova et al. (2020), Di Liello et al. (2019), Jung et al. (2019), Mueggler et al. (2023), Shie et al. (2023), Sumkhemthong et al. (2021), Evans et al. (2021), Sulimanov et al. (2023)
Identification of cell surface markers	Immunostaining for cell sorting and flow cytometry	Used to identify specific cell types within heterotypic 3D cultures, to study specific oncogenic markers (e.g., PD-L1-positive cells) or identify cellular drug responses such as apoptosis (e.g., using Annexin V-FITC/PI). Changes in cell populations (e.g., immune cells or cancer stem cells (CSC)) can be studied	Jung et al. (2019), Thirusangu et al. (2022), Evans et al. (2021), Sulimanov et al. (2023), Sumkhemthong et al. (2021)
Cellular invasion	Invasion assay	Matrigel alone or mixed with specific drug concentrations have been used to assess spheroid invasion. Imaging with a light microscope is carried out at predetermined time-points	Kumari et al. (2020), Lee et al. (2021), Thirusangu et al. (2022)
Gene and Protein expression analysis	Whole-exome and RNA sequencing	Traditional sequencing extraction and sequencing techniques using dissociated lung cancer spheroids or organoids	Kim S. Y. et al. (2021), Shie et al. (2023)
	Real-time (RT) PCR and multiplexing PCR	Traditional RNA extraction and PCR techniques to identify mutations and drug-targeting responses	Sumkhemthong et al. (2021), Banda et al. (2020)
	Western blotting	Traditional Western blotting techniques applied to extracted proteins from dissociated lung cancer spheroids or organoids	Kim S. Y. et al. (2021), Sumkhemthong et al. (2021), Thirusangu et al. (2022)
Molecular Identification	Novel fluorochrome imaging	An imaging technique designed for NSCLC precision-cut lung slices molecular identification	Azari et al. (2024)
Other parameters	Oxygen and pH distribution	Commercially available dyes based on fluorescence	Shie et al. (2023)
	Glucose uptake	2-NBDG dye fluorescence release	Shie et al. (2023)

### 6.1 Cytotoxicity and apoptosis assays

One of the most widely used techniques in studies involving lung cancer 3D models is flow cytometry. Live cell stains including Calcein acetomethoxy (AM), fluorescein diacetate (FDA) and acridine orange (AO) and dead cell stains such as PI and Ethidium homodimer-1 (EthD-1) are commonly used to analyse cell viability in lung spheroids and organoids (Ivanova et al., 2020). Annexin V-FITC/PI is used to identify apoptotic cells by the binding of annexin V-FITC to phosphatidyl serine moieties on the cell membranes (green) and counterstaining the nucleus (red) with PI (Jung et al., 2019; Sumkhemthong et al., 2021). Cleaved caspase-3 anti-body has been used to visualize apoptotic activity in lung cancer 3D models using immunofluorescence microscopy. DAPI is commonly used as cell nuclear counterstain (Jung et al., 2019). Other brand-specific live/dead cell dyes are often used to analyse live/dead proportions in these 3D cultures (Lee et al., 2019; Jung et al., 2019; Evans et al., 2021). 3D cultures are dissociated for staining and analysis by flow cytometry which is often combined with fluorescence microscopy in order to visually observe fluorescent cells or measure average intensity using software such as ImageJ in lung cancer 3D cultures (Kumari et al., 2020; De Ridder et al., 2022; Ilhan-Ayisigi et al., 2020). Confocal microscopy is commonly used for fluorescent visualization in 3D (Banda et al., 2020; Shie et al., 2023; Mueggler et al., 2023). Thirusangu et al. (2022) report the use of trypan blue extrusion counting as a measure of cell viability in lung cancer spheroids.

Several colorimetric techniques can be used to study cytotoxicity. The 3-(4,5-dimethylthiazol-2-yl)-2,5-diphenyltetrazolium bromide (MTT) assay has been reported to assess cell viability in lung cancer spheroids. Incubation times with MTT can be extended up till 1 day (Daunys et al., 2021). Spheroids grown in Matrigel were extracted using cold PBS-EDTA solution prior to cell viability analysis (Della Corte et al., 2019; Di Liello et al., 2019). Resazurin-based dyes have also been used to study cell viability in lung cancer spheroids (Shie et al., 2023). Commercially available kits involve a simple time-efficient protocol to assess ATP content in spheroids and organoids via a luminescent-based assay, very often used to quantify cell viability in lung cancer 3D models (Huang et al., 2020; Shi et al., 2020; Thirusangu et al., 2022; Kim et al., 2019; Kim S. Y. et al., 2021; Gendre et al., 2021). Other commercially available kits have been developed to study cytotoxicity via fluorescence emission. The dye penetrates ruptured cell membranes, binds to the DNA and gives off a fluorescent signal, which can be quantified by spectrophotometry and imaged with fluorescence microscopy for up to 72 h (Koudan et al., 2021; Appleton et al., 2021; Barbosa et al., 2021). The release of lactate dehydrogenase (LDH) from cells is used to identify cytotoxicity. Many studies employ LDH assays to investigate drug-induced cytotoxicity in different types of lung cancer 3D models (Thirusangu et al., 2022; Barbosa et al., 2021).

### 6.2 Morphological and mechanical analysis

ImageJ is widely used among scientists to measure spheroid and organoid morphological parameters through light microscopy (Jung et al., 2019). An open-source ImageJ plugin, SpheroidJ, has been released for the analysis of spheroid morphological characteristics (Lacalle et al., 2021). AnaSP (Analysis of SPheroids) has been developed with the intention of facilitating the acquisition of spheroid quantitative morphological data through 2D brightfield imaging. This open-source software allows for automatic, precise and accurate spheroid segmentation which has been optimized in versions *2.0* and above, enabling high-throughput analysis of morphological parameters (Piccinini, 2015; Piccinini et al., 2023). However, till now, no studies involving lung cancer 3D models have been reported to use AnaSP software. These morphological analyses can be used to assess changes in spheroid and organoid growth kinetics (e.g., diameter or cell scattering) with time (Jung et al., 2019; Gendre et al., 2021; Sumkhemthong et al., 2021; Banda et al., 2020; Ilhan-Ayisigi et al., 2020; Mueggler et al., 2023).

Stiffness, as a mechanical property of biological tissues, is a crucial parameter that influences cellular behaviour, including cell proliferation, differentiation, migration, and apoptosis. Atomic force microscopy and has been used to study drug effects on LCO stiffness (Sen et al., 2023).

### 6.3 Immunohistochemistry and immunofluorescence

Several intra-tumoural parameters can be studied via immunohistochemistry (IHC) of tissue slices. Spheroids can be fixed in 4% formaldehyde or 4% paraformaldehyde, with fixing times varying between different protocols. Cryosectioning or paraffin-embedded (FFPE) sectioning, followed by haematoxylin and eosin (H&E) staining can be used to study micro-tissue anatomy of lung cancer 3D cell cultures, before and after drug treatments (Kumari et al., 2020; Charlet-Faure et al., 2023; Yoshimoto et al., 2023; Mueggler et al., 2023; Kim S. Y. et al., 2021).

Immunostaining of lung 3D cultures with specific antibodies allows for spatial visualization of cells, proteins or targets of interest, both on the surface and inside of the 3D structures allowing for understanding morphological and molecular alterations with drug treatments (Daunys et al., 2021). Anti-EpCAM antibodies can used to identify tumour cells within heterotypic cell populations. In tumour cells not expressing EpCAM other staining methods can be used such as Hoechst staining followed by nuclear morphological analysis. Stromal cell viability can be assessed through EpCAM-negativity (Ivanova et al., 2020). To mention another few, cytokeratin, F-actin, E-cadherin, vimentin and Ki-67 antibodies are commonly used to study structural features or changes in marker expression in lung cancer 3D cultures for pharmacological purposes (Di Liello et al., 2019; Mueggler et al., 2023; Shie et al., 2023).

Cell sorting and flow cytometry provide a platform for the identification of cell surface markers of dissociated 3D cell cultures, most commonly spheroids and organoids (Thirusangu et al., 2022). Flow cytometry is indispensable when dealing with heterotypic cultures for the study of different cell types and sub-populations, such as the identification and characterisation of M1 and M2 TAMs (e.g., through CD64 and CD206 staining, respectively) or CSC markers (e.g., CD133) and the study of specific oncogenic markers (e.g., PDL-1-positive tumour cells) (Evans et al., 2021; Sumkhemthong et al., 2021; Sulimanov et al., 2023).

### 6.4 Spheroid invasion assay

Being a major culprit responsible for the high mortality of lung cancers, tumour invasion directly impacts our understanding of the lung cancer progression, treatment response and patient outcomes. Spheroids or organoids can be embedded in Matrigel mixed with concentrated drug solutions and imaged using light, phase contrast or confocal microscopy over pre-determined time-points to analyse distance or area of invasion as variables to investigate drug effects on tumour cell invasion (Kumari et al., 2020; Lee et al., 2021; Thirusangu et al., 2022).

### 6.5 Gene and protein expression analysis and other molecular identification techniques

Several traditional analytical techniques can be used to analyse expression profiles and specific molecules in lung cancer 3D culture cells. Western blotting on proteins extracted from lung cancer spheroids and organoids was carried out to investigate drug-induced alterations in protein expression (Sumkhemthong et al., 2021; Thirusangu et al., 2022). Whole-exome and RNA sequencing were also conducted on lung cancer 3D models (Kim S. Y. et al., 2021; Shie et al., 2023). Recently, Azari et al. (2024) developed and validated a fluorochrome imaging technique for NSCLC PCLS for molecular identification. RNA extraction of lung cancer spheroids followed by real-time (RT) PCR has been described by Sumkhemthong et al. (2021). Banda et al. (2020) have reported the use of multiplexing PCR for mutational analysis in lung cancer PDOs.

### 6.6 Assessing other parameters

Shie et al. (2023) report the use of commercially available dyes to measure oxygen and pH distribution throughout lung cancer spheroids. Changes in glucose uptake of spheroids exposed to treatment have also been studied using 2-NBDG dye fluorescence release.

## 7 3D cell culture models in drug development

**FIGURE 6:**
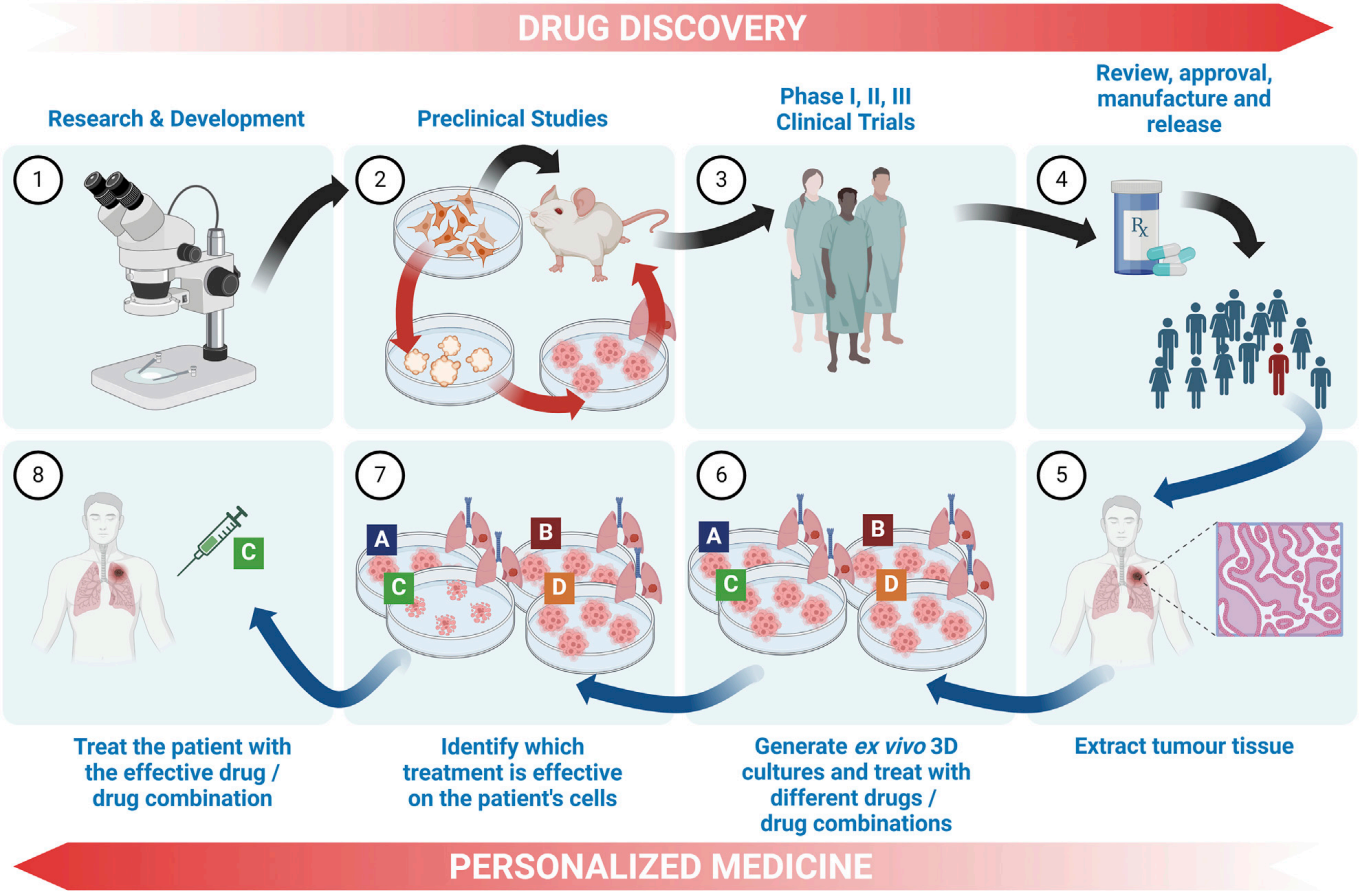
The contribution of 3D cell cultures to drug discovery and personalized medicine in lung cancer. *Drug discovery:* The drug discovery workflow involves (1) research and development, and moves on to (2) preclinical cell culture and animal model testing, (3) clinical trials and (4) approval, release and post-marketing surveillance. 3D cell culture models bridge the gap between pre-clinical 2D cell culture and animal studies, thus reducing the number of animals required and reducing the risk of therapeutic failure and/or toxicity in the clinical stages. *Personalized medicine:* 3D cultures can contribute to personalized medicine by (5) extracting tumour tissue from a patient, (6) generating *ex vivo* 3D cultures and exposing them to a panel of chemotherapeutic drugs or drug combinations (7) identifying the drug or drug combination that is most effective for the patient’s cells, and (8) treating the patient with the identified optimum chemotherapy. (Legend: Black arrows - normal drug development workflow; red arrows - introduction of 3D models into pre-clinical stages; blue arrows - workflow for 3D cell culture applications in personalized medicine).

The application of 3D cell culture models in drug screening and toxicity testing represents a significant enhancement over traditional methods. While animal models have been the gold standard for pre-clinical *in vivo* studies, they often fail to accurately predict human responses due to species-specific differences. 3D cell cultures bridge this gap by offering a more human-relevant model, and especially when used in tandem with animal studies, they provide a more comprehensive assessment of a drug’s pharmacological profile before progressing to clinical trials (Figure 6). Furthermore, this combined approach also enables the use of less animals during the pre-clinical drug development phases, as well as reduces the likelihood of late-stage failures in clinical trials. The fidelity of such a system can be further enhanced through the application of co-culture approaches.

The incorporation of 3D cultures in drug development, presents several challenges. The diverse types of 3D cultures often presents a dilemma as to which particular model best simulates *in vivo* conditions for the generation of clinically relevant data for a specific drug or drug class. Furthermore, industrial drug development requires systems that can be used in high-throughput modalities. Such challenges have often hampered their use for large scale primary screening. These have consequently often limited their applications to smaller scale experiments and validation involving single end-point measurements, towards the middle to lower end of the pre-clinical drug development workflow (Booij et al., 2019).

Specifically, 3D models need to be (a) scalable to industrial level requirements. (b) automatable to enable inclusion into a large-scale drug development workflow, and (c) reproducible in order to ensure assay precision and validity of data obtained from testing of different compounds. Furthermore, up-scalability and automation also need to be applicable to any imaging and assay measurements required as experimental endpoints. While automated liquid handling techniques are standard procedures widely adopted in the general industry, these tend to perform poorly when using highly viscous materials such as natural hydrogels. This is further compounded by the inter-batch variability in protein content and composition of these naturally-derived products, often influencing their temperature-dependent consistency and cellular responses (Booij et al., 2019). Notwithstanding such disadvantages, gel-based scaffolds still remain the 3D cell culture models which are most functionally adapted to large-scale drug screening. Although liquid-based models, such as those generated by hanging drop or ULA plates, are easier to automate, the lack of ECM proteins in such models influences their ability to enable the remodelling required to support normal cell adhesion, growth, differentiation and cell-cell communication (Cushing and Anseth, 2007). The choice between natural and synthetic hydrogel use in such systems remains a matter for debate. The former demonstrate higher *in vivo* mimicry but poorer batch-to-batch reproducibility, while the latter are less accurate at mimicking *in vivo* tumours, but show higher batch-to-batch consistency and therefore higher experimental precision.

The most promising gel matrices are synthetic hydrogels to which are added functional synthetic peptides which mimic those in natural ECM, such as peptides that mimic fibronectin, laminin–integrin interaction sites and collagen-derived adhesive peptides. Moreover, proteolytically degradable domains can be included in these protein mimics, in order to further allow complex 3D cellular behaviour (Gjorevski et al., 2016; Miller et al., 2010). The synthetic nature of these products ensures batch consistency, however there is still no universal agreement on the profile and nature of functional peptides that should be used in order to best mimic the ECM of different tumours.

High-throughput automated systems capable of handling assay execution for 3D models, especially assays involving histological analysis and imaging, have also proven to be a formidable challenge. Conventional imaging techniques are inadequate to capture 3D data and confocal or multiphoton microscopy is necessary to study the internal micro-tumour core. Moreover, the actual thickness of scaffold-based models, also presents problems with chemical-based endpoint assays, since it is difficult to ensure penetration of assay compounds to the core of the model.

In order to mitigate this, Wu et al. developed an electronic biosensor detection and recording approach to study drug screening in a panel of lung cancer spheroids derived from H549, H1299 and H460 cell lines. Their approach was to use electrical impedance sensing to record the reduction in spheroid size upon the application of anticancer drugs under study. The authors identified a synergistic effect of cisplatin plus etoposide on A549 spheroids. The authors further developed a multi-organ spheroid system, using A549, HL-1 and HepG2-based spheroids, cultured on the same multi-well plate, with interconnected wells such that the different spheroids share the same flow path. This model was used to study the toxicity of lung cancer chemotherapeutic agents on the heart and liver, with simultaneous recording of impedance in multiple wells. This is a promising pathway for high-throughput sensing and recording of drug effects which may be up-scaled to industrial-based anti-cancer drug screening (Wu et al., 2018).

Microfluidic systems offer a promising future for integration into drug development, since they satisfy the requirements of automation and scalability. Moreover, they are largely based on liquid handling engineering technologies, offering precise control of active fluidic transport and content, and making them more amenable to high-throughput adaptation. The possibility of increasing complexity by modelling interactions between different organs on the same chip, sharing the same circulatory environment, greatly increases the potential for these models to serve as industry-level drug screening and testing platforms for new anti-cancer drugs.

## 8 3D cell culture models and personalized medicine

The National Cancer Institute of the NIH has defined cancer-related personalized (or precision) medicine as an approach that “uses specific information about a person’s tumour to help make a diagnosis, plan treatment, find out how well treatment is working, or make a prognosis” (National Cancer Institute, 2024). It aims to address issues regarding the underlying inter-patient tumour heterogeneity by establishing therapeutic procedures that are tailored to that individual’s unique biochemical, physiological, genetic and behavioural profile (Goetz and Schork, 2018). Within this framework, it is necessary to study live patient material from tumour biopsies or resected tumour tissue within an *ex vivo* laboratory environment, in order to propose a patient-optimized therapeutic profile (Figure 6). This is particularly useful for rare or aggressive tumours where maximum treatment efficacy within the minimum time is mandatory.

The suitability of 3D cell cultures for personalized medicine has been recently extensively reviewed by El Harane et al. (2023). A key advantage is that patient-tissue derived 3D models carry the same cellular heterogeneity and genomic diversity of the patient’s tumour. Such models afford the ability to interrogate tumour responses to targeted therapies and chemotherapeutic agents within a relevant patient-dependent context, thereby facilitating the identification of patient-specific drug sensitivity and resistance. The models need to mimic the *in vivo* tumour with enough accuracy in order to enable *ex vivo* data to be clinically translatable to a patient’s personalized treatment profile. This shifts the emphasis from the industry-required high-throughput and automation, to critical requirements of accurate model mimicry, and clinically translatable treatment data within an actionable timeframe (Cantrell and Kuo, 2015).

PDO models currently occupy centre stage as lung cancer models suitable for the study of personalized chemotherapies (Fong et al., 2017; Law et al., 2021; Li. Y. et al., 2023; Li H. et al., 2023; Liu et al., 2023; Wang H. M. et al., 2023; Gu et al., 2024). PDOs can be generated in a relatively short time, and can be genetically profiled in order to identify tumour-dependent acquired mutations (Hu Y. et al., 2021). There is also ample evidence to show that the cellular composition and protein expression profiles and mutation heterogeneity of the patient’s tumour are retained in PDO models (Kim et al., 2019). Banda et al. showed that chemotherapy-driven enrichment of specific mutant sub-populations of patient-derived 3D models, also mimics what occurs *in vivo*. For example, erlotinib-treated spheroids derived from patient lung adenocarcinoma, showed a major increase in *PIK3CA* H1047R mutant subpopulations compared to *BRAF* V600E, *KRAS* G12D, *KRAS* G12V, and *PIK3CA* H1047R mutant cells, which aligns with what happens in patients. Therefore, *ex vivo* cultures not only replicate the tumour composition at the time of biopsy, but also project temporal and treatment-induced changes, in the same manner as occurs in patients (Banda et al., 2020; Taverna et al., 2024).

The reported success rates in the generation of *ex vivo* lung cancer organoids have risen along the years and now generally exceed 75% (Kim S. Y. et al., 2021; Wang M. et al., 2023; Shi et al., 2020; Hu Y. et al., 2021), adding to their suitability for precision medicine. Within NSCLC subtypes, SCC organoids appear to have the greatest variability in success rates between different research groups, which is however still reportedly higher than with other cancers. This places lung cancer as a leading candidate for *ex vivo*-based precision medicine (Liu et al., 2023; Rossi et al., 2022).

It however remains a challenge to obtain lung cancer tumour tissue, unless the patient is scheduled for surgery. Lung cancer biopsies tend to carry risks and may require strong medical justification in order to be carried out. Pleural effusion aspirate may prove to be an alternative suitable source of tumour cells. The generation of 3D organoids from the drained pleural effusion of lung adenocarcinoma patients has been described by Mazzocchi and co-workers, who showed that the generated organoids contained cancer cells with both immune and stromal components including fibroblasts and haematopoietic cells. Such organoids also produced a drug response profile to a cisplatin/pemetrexed combination, carboplatin/pemetrexed combination and crizotinib alone, that was similar to the responses seen in the patients from whom the pleural effusions were derived. This suggests that drained pleural effusion may be a more suitable and accessible source of lung cancer cells for personalized medicine approaches (Mazzocchi et al., 2019; Mazzocchi et al., 2022).

Zhang and co-workers later developed a novel *ex vivo* lung cancer model, termed a lung cancer assembloid, that uses droplet microfluidic technology to replicate the three-dimensional architecture and heterogeneous microenvironment of actual lung tumours. This allows for the encapsulation of TME cells and organoids within microgels, leading to an accurate representation of the tumour’s heterogeneity in terms of cell types, genetics, and responses to drugs. The authors utilized this model to study the potential drug resistance that may occur *in vivo* due to the presence of cancer-associated fibroblasts and other stromal elements, and showed that this model can accurately replicate the clinical outcomes of patients (Zhang et al., 2024).

Challenges which hinder the widespread clinical applications of 3D model-driven personalized therapy for lung cancer remain. The most critical of these is the lack of validated and standardized specific models or protocols that dictate which 3D models should be used for each situation and how such models should be generated. Until there is a general consensus reflected by evidence-based science, applicable to different lung cancer subtypes, there is bound to be data disagreement between different working groups. Furthermore, the inherent complexity and heterogeneity of lung cancer makes it difficult to create *ex vivo* models that are representative of *all* aspects of the disease. This coupled to difficulties in establishing the appropriate culture conditions that support their growth and maintenance while preserving the original tumour characteristics, as well as the variable *ex vivo* model generation success rate reported by different groups, drives some researchers to question the current applicability to precision medicine. More work is needed before attaining a stage where regulatory agencies can be convinced that this is an important step towards improving lung cancer patient treatment efficacy and approve its incorporation into clinical practice.

## 9 Discussion

The generation of effective pharmacological tools for lung cancer remains a major challenge and this is undoubtedly one reason for its long-standing elevated position in the lethal hierarchy of solid tumours. The use of 3D cell culture models in lung cancer pharmacology research represents a paradigm shift in the approach towards understanding and treating this disease. The scientific community, together with policymakers and funding agencies, need to recognize the value of these advanced research tools and continue to support their development. With continued innovation and investment, 3D cell culture models will undoubtedly play an integral role in shaping the future of lung cancer therapy, offering hope for an increased quality of life to millions of patients worldwide.

Research in this area is active and progressing along two lines. The first is the development of further tumour characteristics that can be modelled, such as vascularization and oxygen tension. The second is the improvement of incorrectly modelled components which actually may contribute to false experimental outcomes. For example, most reported spheroids are smaller than 400 μm in diameter, while passive drug diffusion across cell layers reaches a depth of about 200 μm (Shie et al., 2023; Hamilton and Rath, 2019; Bauleth-Ramos et al., 2020). Therefore, small spheroids do not accurately model intra-tumoural gradients and this could be one factor that contributes to the often-reported discrepancies of drug IC_50_ values obtained via 2D and 3D culture studies. We suggest that larger spheroids are more amenable to reproduce similar drug diffusion gradients to those which occur in much larger *in vivo* tumours.

Within the lung cancer research domain, there have been very few reported studies of models which preserve the *in vivo* lung tissue architecture such as PCLS, WTE and OMS. This may be due to the greater technical difficulties in maintaining such models, with the corresponding low outcome success rates. The lung TME is central to the behaviour and response to treatments and its mimicry is essential for translatable experimental outcomes. As mentioned in this review, the use of dECM in conjunction with 3D lung cancer models has been briefly explored. The dECM used in these models was sourced from animals and the application of human lung dECM remains an area of exploration, offering potential new insights into lung tumour biomimicry. In addition, novel innovative technologies, such as the use of micro-beads to simulate the alveolar architecture and therefore model lung tumours developing in the alveolar region, need to be explored more. ALIs offer a significant promise towards modelling airway epithelial architecture, especially multi-layered co-cultured models. This has been evidenced through morphological, transcriptomic and secretory studies which showed ALIs to behave very similarly to an *in vivo* epithelial system (Geles et al., 2016). However, although these have been extensively used for other airway conditions such as inflammatory diseases, they have only been marginally applied in lung cancer studies. ALIs provide a unique system where in addition to disease, they can also model both systemic as well as aerosolized drug administration (Meenach et al., 2016; Movia et al., 2018). This makes them an insufficiently tapped resource for 3D lung cancer models and their increased application can provide new data which is of strong pharmacological relevance.

Drug development efforts have been unable to result in levels of effectiveness which are comparable to those achieved for other common cancers. This is further hampered by the long and costly bench to bedside drug development procedures and the minute fraction of screened compounds that actually successfully filter through to the final clinical testing stage. If 3D lung cancer models are expected to generate tangible time and cost-benefits which can translate to improved therapeutic tools, there are several creases in the system that need to be ironed out. The major one is perhaps the lack of agreement on which 3D model best mimics a specific disease. The plethora of variables in 3D cell culture model setups is likely to take multiple years of research before any convergence of agreement on an optimum model or set of models that yields accurate, reproducible and timely results can be established. The possibility of never being able to model the complete TME factors, including interactions of various cell types, tumour cell heterogeneity, vascularization, intra-tumoural oxygen gradients, drug penetration and the influence of other organ systems, has undeniable implications on the data generated by the model. However, the last decades have provided evidence that event and data modelling in areas such as material science are excellent substrates for *in silico* operation. Digital infrastructures offer the potential of acting in tandem with biological models to create more accurate output models. We propose that this approach could also be applied to biological modelling, in order to electronically compensate for missing or inaccurate components in 3D lung cancer models. Digital enhancement of such biological data can improve human *in vivo* mimicry and push forward its functional applicability to novel drug development.

The precision medicine academic and clinical areas have been greatly overtaken by genomic and transcriptomic-based treatment personalization. Within this context, *ex vivo* laboratory disease modelling is still at its infancy. However, genomics and transcriptomics are more adept at predicting disease outcomes due to germline mutations and less effective at addressing the genetic heterogeneity of acquired mutations in tumours. In this regard, 3D cultures currently offer the best *ex vivo* model for the implementation of personalized medicine and as suggested earlier, are also open to digitally-assisted enhanced modelling in order to direct optimized predictive data.

## 10 Conclusion

In summary, the 3D lung cancer modelling world is rapidly evolving and its profile of application areas will increase as new developments progressively show greater model-to-human translatability. However, higher *in vivo* mimicry is generally also associated with more complex model systems requiring higher cost and technical expertise. A balance between attainment of usable translatable data *versus* cost and time efficiency would likely best drive the way forward. For example, body-on-a-chip microfluidic devices offer excellent academic promise but may be too technically challenging to be implemented within a cost-effective industrial drug development framework. On the other hand, simpler models, such as spheroids, might provide less robust data but are more easily implementable. A balance might be found through the use of computational and perhaps AI modelling systems in tandem with biological models. This would potentially create a complementary digital-biological modelling system that is amenable to a broader application profile with higher throughput and cost efficiency.
